# Metal Coordination Enhances Chalcogen Bonds: CSD Survey and Theoretical Calculations

**DOI:** 10.3390/ijms23084188

**Published:** 2022-04-10

**Authors:** Antonio Frontera, Antonio Bauza

**Affiliations:** Departament de Química, Universitat de les Illes Balears, Crta. de Valldemossa km 7.5, 07122 Palma de Mllorca, Baleares, Spain

**Keywords:** σ-hole interactions, chalcogen bonding, supramolecular chemistry, DFT study, metal coordination

## Abstract

In this study the ability of metal coordinated Chalcogen (Ch) atoms to undergo Chalcogen bonding (ChB) interactions has been evaluated at the PBE0-D3/def2-TZVP level of theory. An initial CSD (Cambridge Structural Database) inspection revealed the presence of square planar Pd/Pt coordination complexes where divalent Ch atoms (Se/Te) were used as ligands. Interestingly, the coordination to the metal center enhanced the σ-hole donor ability of the Ch atom, which participates in ChBs with neighboring units present in the X-ray crystal structure, therefore dictating the solid state architecture. The X-ray analyses were complemented with a computational study (PBE0-D3/def2-TZVP level of theory), which shed light into the strength and directionality of the ChBs studied herein. Owing to the new possibilities that metal coordination offers to enhance or modulate the σ-hole donor ability of Chs, we believe that the findings presented herein are of remarkable importance for supramolecular chemists as well as for those scientists working in the field of solid state chemistry.

## 1. Introduction

Since the beginning of the 21st century, the ability of elements from Groups 13–18 covalently bound to electron withdrawing groups (EWG) to favorably interact with Lewis bases (e.g., lone pair donors, π-systems and anions) has been subject of extensive investigation [1,2,3,4,5,6,7,8,9,10,11,12,13]. It all began with the standardization of the electropositive site to describe the main features of the hydrogen bonding (HB) interaction and from there, it became common to name the noncovalent interactions (NCIs) between nucleophile and electrophile sites (known as σ-holes) by using the name of the group to which the electrophilic atom belongs [14,15]. In this context, the International Union of Pure and Applied Chemistry (IUPAC) have already recommended the terms halogen bond (HaB) [16] and chalcogen bond (ChB) [17] for naming the NCIs encompassing atoms from groups 17 and 18, respectively. Furthermore, a specific name is given to each group, being aerogen or noble gas bonding (NgB, group 18) [12], pnictogen bonding (PnB, group 15), [18,19] tetrel bonding (TtB, group 14) [20], and triel bonding (TrB, group 13) [7]. Furthermore, elements from groups 7, 8, 11 and 12 acting as Lewis acids have recently received the names of matere bonding (MaB, group 7) [21], osme bonding (OmB, group 8) [22], spodium bonding (SpB, group 12) [23] and regium or coinage bonding (CiB, group 11) [24,25,26,27].

The use of σ-holes as an alternative to HB interactions has been reported in many studies belonging to a broad spectrum of fields, such as host-guest chemistry, catalysis, supramolecular chemistry, membrane transport, crystal engineering, etc. [28,29,30,31,32,33,34,35,36,37,38,39,40,41,42,43,44,45]. In addition, comparisons have been made to unveil similarities and differences between σ-hole interactions and the HB in both energetic and geometric characteristics [46,47,48,49,50,51].

Nowadays a remarkable progress has been achieved regarding the supramolecular chemistry of chalcogen bonding, particularly in regulating and fine tuning novel chemical systems for applications in crystal engineering, supramolecular chemistry, catalysis, transport of anions and functional materials [3,9,10,42]. As a common feature among the σ-hole family of interactions, the physical nature of the ChB is mainly based on electrostatics, while dispersion forces, polarization, charge-transfer, orbital delocalization and π-conjugation are also potential contributors to the ChB formation and strengthening [52,53,54,55]. In this context, two key features of the ChB interaction are both the strength and directionality. The former is related to the difference between the sum of van der Waals radii of the interacting atoms (Σ*r*_vdW_ (Ch···A), where A = Lewis base) and the experimental Ch···A distance. The latter is related to the location of a σ-hole (opposite to the R–Ch bond), hence, the strength of the interaction is maximized at a ∠R–Ch···A angle of 180°, representing a measure of the directionality. In a parallel way to other σ-hole based interactions, both the strength and directionality of ChBs mainly depend on several factors:

**Ch atom involved:** Conversely to HB, the σ-hole donor ability varies upon modification of the Ch atom [39,56,57]. In Table 1 are gathered the atomic polarizabilities (α) and van der Waals radii (R_vdW_) of the chalcogen elements from period 2 to 5. As noted, the atomic α value becomes higher from 3.0 a.u. in O to 25.9 a.u. in Te. Interestingly, the difference in the atomic polarizability between O and S is noticeable (around 4 times higher for S), whilst the variations between S and Se or Se and Te are of lesser magnitude.

**Figure 1:**
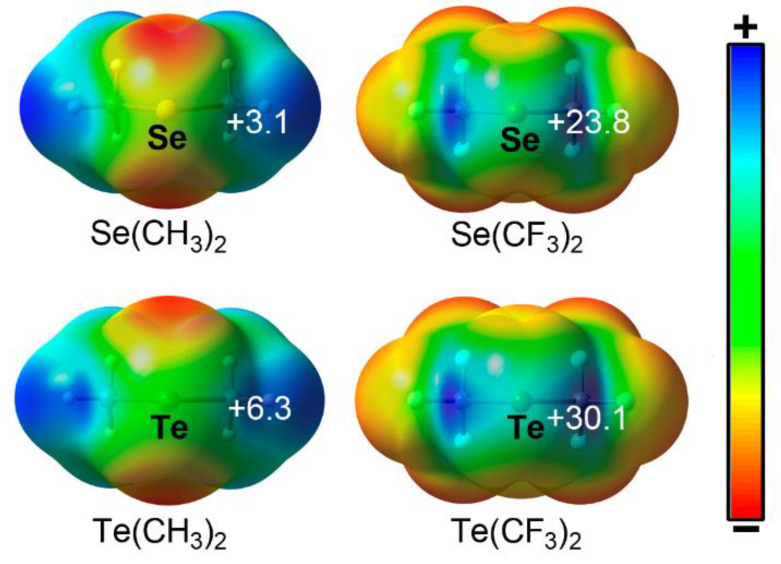
MEP surfaces (MP2 [65]/def2-TZVP [66] level of theory) of the Ch(CH_3_)_2_ and Ch(CF_3_)_2_ (Ch = Se and Te) molecules. The energy values at concrete points of the surface are given in kcal/mol (0.001 a.u.). The calculations have been performed by means of the Turbomole 7.0 software (Karlsruhe, Germany) [67].

**R groups attached to the Ch atom:** The substituents (R) effect to the Ch···A bond features have been analysed from a theoretical perspective [58,59,60,61,62], evidencing that EWG enhance the strength of the ChB through the formation of larger and deeper σ-hole(s). Conversely, the use of electron-donating groups (EDG) resulted in a weakening of the interaction. Both types of substituents have been used to correlate structure-property functions, as well as a source of stabilization of the secondary coordination sphere in metal complexes. Figure 1 shows several Molecular Electrostatic Potential (MEP) surfaces of the –CH_3_ and –CF_3_ substituted Selenium and Tellurium derivatives. As noted, two σ-holes are located on each Ch atom on the extension of the C–Se and C–Te bonds and their MEP values become more positive ongoing from Se to Te, in agreement with the atomic polarizabilities discussed above. Moreover, the use of strong EWG (e.g., –CF_3_) increases the potential of the σ-hole, thus enhancing the σ-hole donor ability of the Ch atom and strengthening the interaction. Finally, using EDG as substituents of the Lewis base can also enhance the ChB interaction by increasing the nucleophilicity of the electron rich moiety [63,64].

**Interacting partner (A):** A variety of classical nucleophile species (e.g., lone pair bearing chalcogen, pnictogen or halogen atoms), including metal ions with available *d*z^2^ (Rh^+^, Ni^2+^, Pt^2+^, Pd^2+^) or *d*x^2^ − y^2^(Au^+^) orbitals can act as electron donor moieties to form strong ChBs [68,69,70,71,72,73,74,75]. Moreover, in a similar fashion to aromatic π-systems, the chelate ring has also been observed to participate as a ChB acceptor in intermolecular ChBs [76].

**Figure 2:**
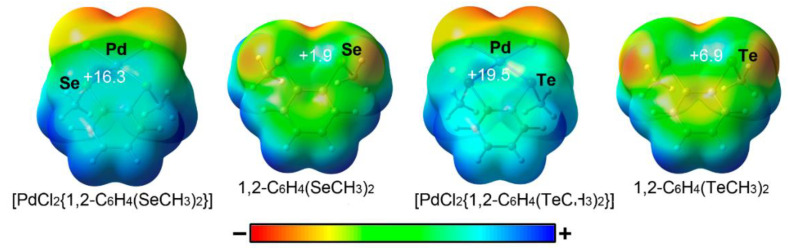
MEP surfaces of [PdCl_2_{1,2-C_6_H_4_(ChCH_3_)_2_}] and 1,2-C_6_H_4_(ChCH_3_)_2_ (Ch = Se and Te) molecules. The energy values at concrete points of the surface are given in kcal/mol (0.001 a.u.).

In this article, we illustrate several X-ray structures to highlight that ChBs in metal complexes can be used as an effective tool in crystal engineering and in the generation of functional materials. Particularly, we have focused our attention on a divalent Ch moiety coordinated to a metal center, which certainly increases the R–Ch σ-hole, leading to new opportunities to use ChBs as a supramolecular tool in coordination chemistry [77,78]. To illustrate this idea, two examples have been selected (see Figure 2), where a bidentate Se/Te ligand is coordinated to a Pd^2+^ ion in addition to two chloride ions in a square planar geometry. Upon coordination, there is one Se/Te σ-hole still available to undergo ChB interactions, which exhibits an enhanced electrostatic potential (up to 14 kcal/mol) compared to the uncoordinated ligand.

**Scheme 1:**
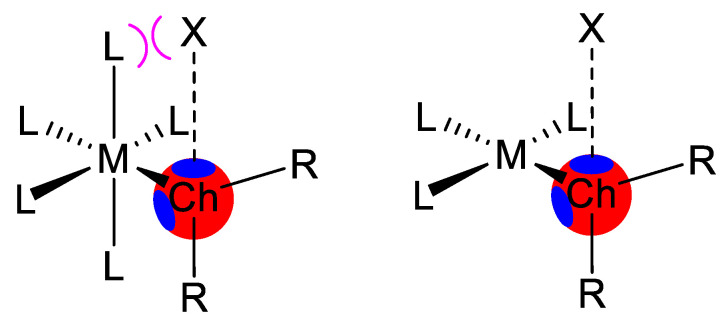
Schematic representation of a ChB interaction in a Ch-coordinated octahedral (**left**) and square planar (**right**) complex.

In this context, the Cambridge Structural Database (CSD) [79] has been inspected and a series of selected examples where Se or Te atoms coordinated to square planar metal coordinated behave as an electrophilic center (chalcogen bond donor) have been discussed. In a parallel way to X-ray analysis, the presence of intermolecular chalcogen bonding in metal organic complexes has been also analyzed from a theoretical perspective. The selection of square planar complexes instead of octahedral is to prevent steric effects that may hamper the formation of ChB interactions, as represented in Scheme 1. We believe this review article will attract the attention of both theoreticians and experimentalists to investigate the ChBs in coordination compounds, which can be used as a new synthon in crystal engineering and improve the functional properties of materials.

## 2. Computational Methods

Calculations regarding the supramolecular complexes have been carried out at the PBE0 [80,81]-D3 [82]/def2-TZVP [66] level of theory using the program Turbomole 7.2 (Karlsruhe, Germany) [67]. The binding energy values (ΔE_BSSE_) were calculated as the energy difference between the optimized structures of the complex and isolated monomers following the supermolecule approximation (ΔE_complex_ = E_complex_ − E_monomerA_ − E_monomerB_). Using the Gaussian 16 software [83], the MEP surfaces of compounds Ch(CH_3_)_2_, Ch(CF_3_)_2_, [PdCl_2_{1,2-C_6_H_4_Ch(CH_3_)_2_}] and 1,2-C_6_H_4_Ch(CH_3_)_2_ (Ch = Se and Te) have been computed at the MP2 [65]/def2-TZVP level of theory.

To compute the contribution of the Chalcogen bond contacts (in kcal/mol) the following equations were used [84]:

E_int_ = 0.375 × V(r) − 0.5655 (for those ChBs involving Se)

E_int_ = 0.556 × V(r) + 0.6445 (for those ChBs involving Te)

Finally, the Bader’s “Atoms in molecules” theory [85,86] has been used to analyze and describe the interactions discussed in this work using the AIMall calculation package [87]. The PBE0/def2-TZVP level of theory was used for the wavefunction analysis as well as for the NBO charge analysis (also using Gaussian-16 software).

## 3. Results and Discussion

### 3.1. CSD Search

We have inspected the CSD and found 103 structures where either divalent Se or Te is coordinated to square planar metal centers, which were in all cases Pt or Pd. No examples with other typical square planar metals like Ni and Rh were found. Remarkably, in 73 out of 103 structures, we detected the existence of ChB interactions, using the following geometric criteria: Ch···X distance shorter than the sum of van der Waals radii plus 0.2 Å (ΣR_vdw_ + 0.2) and ∡ R–Ch···X greater than 160 degrees (X = O, C, Pt, Pd, Cl and I). Table S1 compiles the CSD reference codes, geometric features and donor-acceptor atoms. Most of the hits are observed for Se (40 structures) and chloride as electron donor (52 structures). Other electron donors are Br, I, O and C-atoms belonging to electron rich aromatic rings. Interestingly, we have also observed some structures where it is the metal center that acts as electron donor (3 for Pd and 3 for Pt). The geometric features, energies and characterization of the ChBs are provided in the following sections.

### 3.2. Selenium Derivatives

#### 3.2.1. Oxygen as Electron Donor

**Figure 3:**
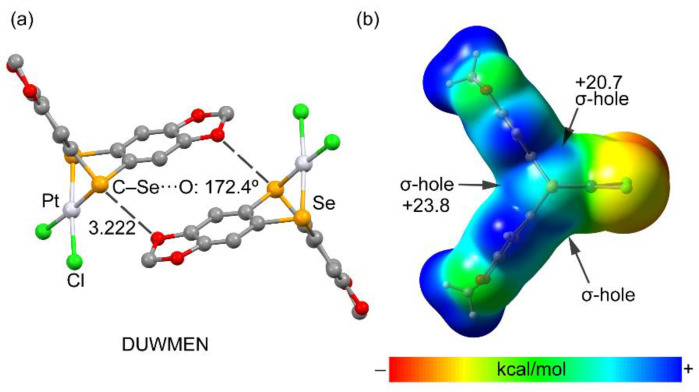
(**a**) Partial view of refcode DUWMEN structure. Distance in Å. H-atoms omitted for clarity. (**b**) MEP surface (isodensity 0.001 a.u.) of DUWMEN. The energies at the σ-holes are indicated in kcal/mol.

Figure 3a shows a self-assembled dimer that is formed in the solid state of dichloro-(2,3:7,8-bis(methylenedioxy)-selenanthrene-Se,Se′)-platinum(II) (refcode DUWMEN [88]). It can be observed that one O-atom of the methylenedioxy group is located opposite to the C–Se bond, thus forming a ChB. The MEP surface (Figure 3b) of this compound reveals the existence of three σ-holes opposite to the three covalent bonds, including the coordination Pt–Se bond. It can be observed that the intensity of the σ-hole opposite to the C–Se bond is large, which is quite unexpected considering the electronegativity of carbon (C–Se bond is not much polarized). This is explained by the coordination of Se to the Pt that increases the Lewis acidity of the Se-atom. It can be also observed that the approximation of the O-atom to the Se could not be possible for a hypothetical octahedral complex due to the presence of an apical ligand.

**Figure 4:**
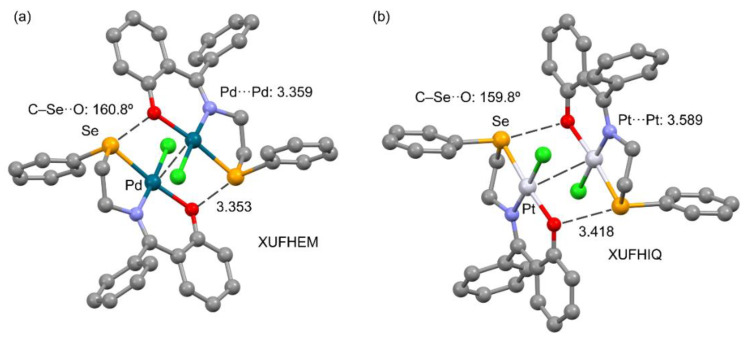
Partial views of the refcodes XUFHEM (**a**) and XUFHIQ (**b**) structures. Distances in Å. H-atoms omitted for clarity.

Two additional examples showing Ch···O contacts with a clear structure directing role are shown in Figure 4. Both complexes present the same ligand [2-(phenyl((2-(phenylselanyl)ethyl)imino)methyl)phenolato], coligand (chloride) and the difference resides in the transition metal (Pd for XUFHEM [89] and Pt for XUFHIQ [89]). Both structures form very similar self-assembled dimers in the solid state where two symmetrically equivalent Se···O contacts are established. Both dimers also present metal···metal (M···M) interactions with distances shorter than 3.45 Å. The Se···O distances are shorter in XUFHEM (Figure 4a) structure, likely influenced by the shorter Pd···Pd distance. The directionality of the ChBs is worse (with respect to linearity) than the observed in DUWMEN structure (Figure 4b), which is also due to the restriction imposed by the M···M interaction.

#### 3.2.2. Halogen as Electron Donor

**Figure 5:**
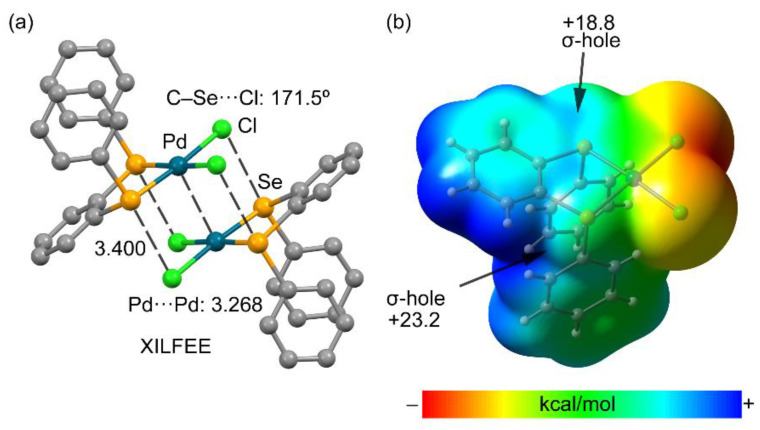
(**a**) Partial view of refcode XILFEE structure. Distances in Å. H-atoms omitted for clarity. (**b**) MEP surface (isodensity 0.001 a.u.) of XILFEE. The energies at the σ-holes are indicated in kcal/mol.

As commented above, most hits from the CSD search present an halogen atom as electron donor, basically because chloride is widely used in the synthesis of Pd and Pt complexes. Figure 5a highlights a self-assembled dimer that is formed in the solid state of (1,2-bis(phenylseleno)benzene)-dichlorido-palladium(II) (refcode XILFEE [90]), selected as representative X-ray structure with Se···Cl contacts. It can be observed that the chloride ligands are located opposite to the C–Se bonds, thus forming four concurrent ChBs and one Pd···Pd contact. The MEP surface of this compound (see Figure 5b) reveals the existence of two σ-holes opposite to (i) one C–Se covalent bond and (ii) to one Pt–Se coordination bond. The second C–Se σ-hole is buried by the large negative belt of Cl. It can be observed that the MEP value at the σ-hole opposite to the Pd–Se bond is larger likely due to the contribution of the nearby C–H bond.

**Figure 6:**
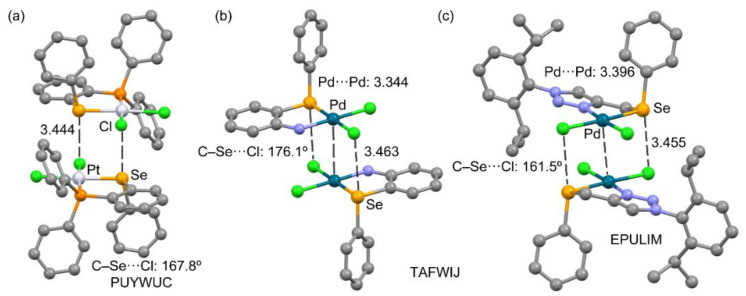
Partial views of the X-ray structures of PUYWUC (**a**), TAFWIJ (**b**) and EPULIM (**c**). Distances in Å. H-atoms omitted for clarity.

Three additional examples showing Ch···Cl contacts are represented in Figure 6, in all cases the adducts are self-assembled dimers. In the dimer of dichloro-(diphenyl(2-(phenylselanyl)phenyl)phosphine)-platinum (PUYWUC [91], Figure 6a), the Pt···Pt interaction is not established and the formation of the dimer is dominated by ChBs. In the other two structures (refcodes TAFWIJ [92], Figure 6b and EPULIM [93], Figure 6c), Pd···Pd interactions coexist with the Se···Cl contacts. Among them, TAFWIJ structure is the one exhibiting the highest directionality (∡C–Se···Cl = 176.1°) and the shortest M···M distance.

#### 3.2.3. Carbon as Electron Donor

**Figure 7:**
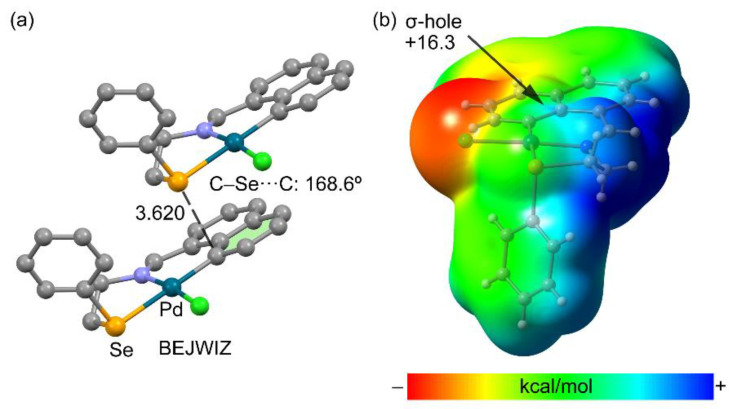
(**a**) Partial view of refcode BEJWIZ structure. Distance in Å. H-atoms omitted for clarity. (**b**) MEP surface (isodensity 0.001 a.u.) of BEJWIZ. The energies at the σ-holes are indicated in kcal/mol.

Figure 7a shows a dimer extracted from an 1D supramolecular assembly that propagates in the crystal packing of chloro-[8-({[2-(phenylselanyl)ethyl]imino}methyl)naphthalen-1-yl]-palladium(II) complex (refcode BEJWIZ [94]). The C-atom bonded to the Pd is located opposite to the C–Se bond, thus playing the electron donor role in this ChB interaction, which is facilitated by the anionic nature of this C-atom. The MEP surface (Figure 7b) shows a small σ-hole opposite to the C–Se bond that merges with the large blue region under the influence of the methylene group.

**Figure 8:**
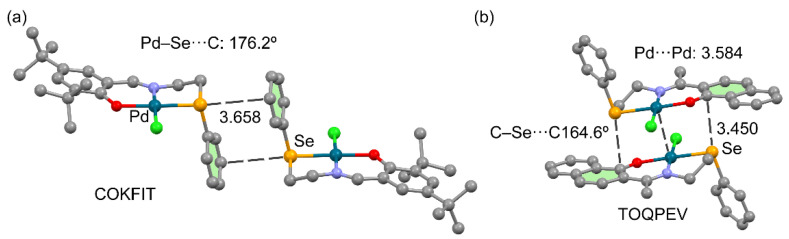
Partial views of the X-ray structures of refcodes COKFIT (**a**) and TOQPEV (**b**). Distances in Å. H-atoms omitted for clarity.

Other two examples of X-ray structures exhibiting Se···C contacts, where the electron donor C-atom belongs to an aromatic system, are given in Figure 8. It is interesting to highlight that for chloro-(2,4-di-*t*-butyl-6-(((2-(phenylselanyl)ethyl)imino)methyl)phenolato)-palladium(II) structure (Figure 8a, refcode COKFIT [95]), the self-assembled dimer does not present M···M interactions and the C-atom is located opposite to the Pd–Se bond. Therefore, in this compound, the third σ-hole at the Se-atom that emerges upon Pd-complexation is responsible for the formation of the dimers. In this example, the ChBs are the only forces governing the assembly. For the other example (TOQPEV [96], Figure 8b), in addition to the ChBs opposite to the C–Se bonds, an M···M interaction is established further supporting the formation of the self-assembled dimer. Curiously, the Pd–Se···C ChBs (dimer of COKFIT) are more directional than the C–Se···C (dimer of TOPQEV). To our knowledge, there are not previous examples in the literature of M–CH···X ChBs, where the electron rich atom is opposite to a M–Ch bond. This interesting and unexplored topic deserves further investigation by the scientific community.

#### 3.2.4. Metal as Electron Donor

**Figure 9:**
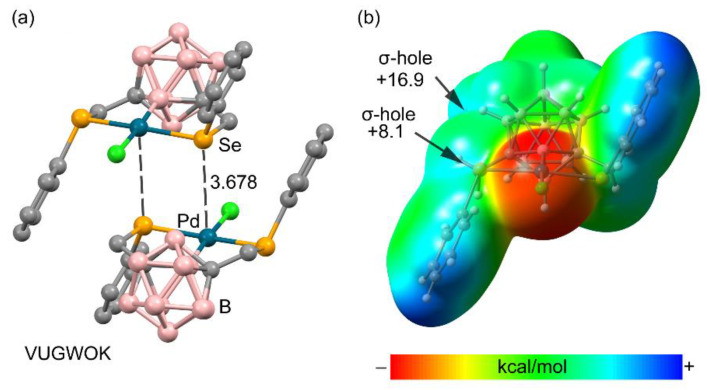
(**a**) Partial view of refcode VUGWOK structure. Distance in Å. H-atoms omitted for clarity. (**b**) MEP surface (isodensity 0.001 a.u.) of VUGWOK. The energies at the σ-holes are indicated in kcal/mol.

The role of metal centers in *d*^8^ or *d*^10^ configuration as electron donors has been recently analyzed for halogen bonding interactions [97]. However, similar investigations for chalcogen bonding are unprecedented. In this section, we show several examples where Pt or Pd act as electron donors in ChBs. Figure 9a shows a self-assembled dimer observed in the solid state of refcode VUGWOK [98]. The Pd is located opposite to the C–Se bond, thus acting as electron donor in the ChB interaction. The MEP surface (Figure 9b) shows a small σ-hole opposite to the Pd–Se bond (+8.1 kcal/mol) and a more intense one opposite to a C–Se bond (+16.9 kcal/mol).

**Figure 10:**
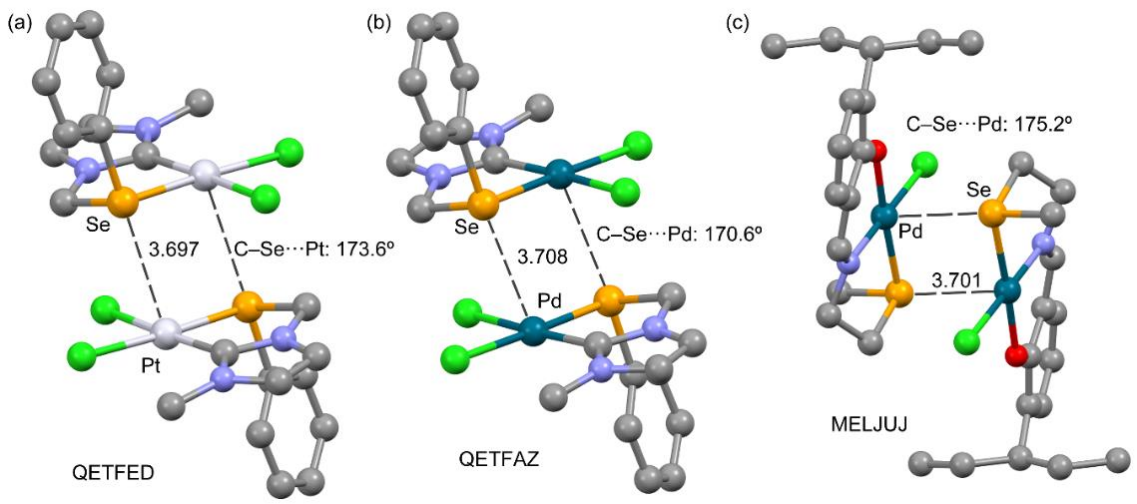
Partial views of the X-ray structures of refcodes QETFED (**a**), QETFAZ (**b**) and MELJUJ (**c**). Distances in Å. H-atoms omitted for clarity.

Other three interesting examples of X-ray structures exhibiting Se···M (M = Pt, Pd) are depicted in Figure 10. It is worthy to comment that the C–Se···M angle is in all cases higher than 170°, thus confirming the strong directionality of the interaction and that the nucleophilic metal center (filled *d*_z_^2^ orbital) is pointing to the σ-hole opposite to the C–Se bond. For the three selected examples (QETFED [99] (Figure 10a), QETFAZ [99] (Figure 10b) and MELJUJ [100] (Figure 10c)) the distances are similar (around 3.7 Å). Finally, in Table 2, the list of CSD codes including compound names and formulas of the Se involving dimers is given.

### 3.3. Tellurium Derivatives

In case of tellurium complexes, the CSD search shows that there are no examples involving oxygen as electron donor. In most of the hits, the electron donor is a halogen atom (Cl, Br or I), a π-system or a metal center (Pd and Pt) as discussed in the following sections.

#### 3.3.1. Halogen as Electron Donor

**Figure 11:**
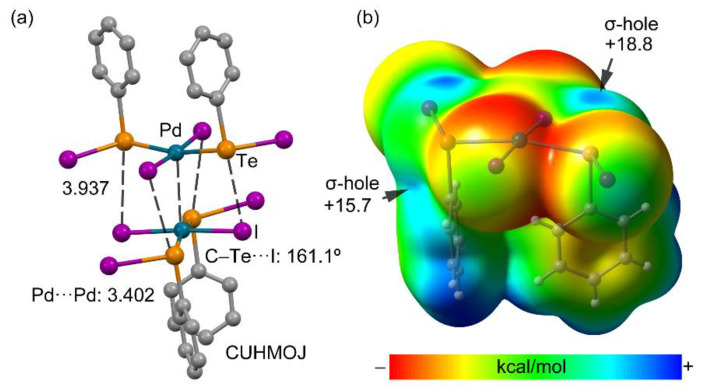
(**a**) Partial view of refcode CUHMOJ structure. Distances in Å. H-atoms omitted for clarity. (**b**) MEP surface (isodensity 0.001 a.u.) of CUHMOJ. The energies at the σ-holes are indicated in kcal/mol.

Figure 11a highlights a self-assembled dimer that is formed in the X-ray packing of bis(benzenetellurenyl iodide)-di-iodo-palladium(II) (refcode CUHMOJ [101]), selected as representative structure showing Te···I contacts. It can be observed that the iodide ligands are located approximately opposite to the C–Te bonds (∡C–Te···I = 161.1°), thus forming four concurrent ChBs and one Pd···Pd contact (3.402 Å). The MEP surface of this compound (see Figure 11b) reveals the existence of two σ-holes opposite to (i) one C–Te covalent bond and (ii) to a Pd–Te coordination bond. The third σ-hole (expected opposite to the I–Te bond) is completely covered by the large and negative belt of I. It can be observed that the MEP value at the σ-hole opposite to the Pd–Te bond is smaller (+15.7 kcal/mol) than that opposite to the C–I bond (+18.8 kcal/mol).

**Figure 12:**
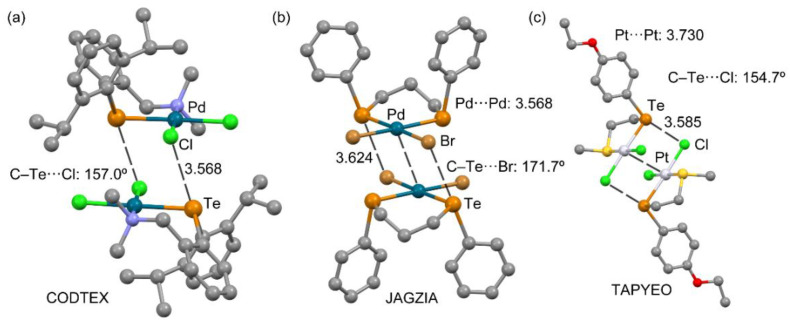
Partial views of the X-ray structures of CODTEX (**a**), JAGZIA (**b**) and TAPYEO (**c**). Distances in Å. H-atoms omitted for clarity.

Three additional examples showing Te···Cl, Br contacts are represented in Figure 12, where all three form self-assembled dimers in the solid state that are relevant in the crystal packing. In the dimer of dichloro-[1-(2-{[2,6-di-isopropylphenyl]tellanyl}phenyl)-N,N-dimethylmethanamine]-palladium(II) (CODTEX [102]), the M···M interaction is not established and the dimer formation is basically dominated by Te···Cl ChBs that are less directional (157°) compared to those previously described for Se (Figure 12a). It is known that the directionality of ChBs decreases on going from S to Te because the size of the σ-hole is larger in the more polarizable Ch atoms. In the other two structures represented in Figure 12 (refcodes JAGZIA [103] (Figure 12b) and TAPYEO [104] (Figure 12c)), Pd···Pd and Pt···Pt interactions coexist with the Te···Br and Te···Cl contacts, respectively. JAGZIA structure is the one with higher directionality (∡C–Te···Br = 171.7°) and shorter M···M distance (3.568 Å) whilst TAPYEO exhibits longer M···M distance (3.730 Å) and smaller angle (∡C–Te···Cl = 154.7°).

#### 3.3.2. Carbon as Electron Donor

**Figure 13:**
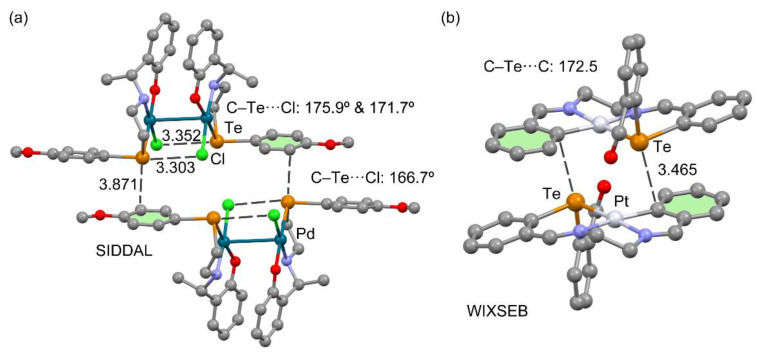
Partial views of the X-ray structures of refcodes SIDDAL (**a**) and WIXSEB (**b**). Distances in Å. H-atoms omitted for clarity.

We have found only two structures in the database showing Te···C interactions, which are represented in Figure 13, refcodes SIDDAL [105] and WIXSEB [106]. In the former the chloro-(2-(1-((2-((4-methoxyphenyl)tellanyl)ethyl)amino)ethyl)phenolato)-palladium(II) forms discrete dimers with short Pd···Pd distance (3.203 Å) reinforced by short and directional Te···Cl contacts (see Figure 13a) using the σ-hole opposite to the C_Aromatic_–Te bond. In addition, these dimers self-assemble via the formation of ChBs using the σ-hole opposite the C_aliphatic_–Te bond where the electron donor C-atom belongs to the aromatic system, with longer distances (3.871 Å) and smaller angles (∡C–Te···C = 166.7°). In the other example (WIBSEX [106] (Figure 13b)) only one type of ChB is established with shorter Te···C distance (3.465 Å) and better directionality (∡C–Te···C = 172.5°) compared to the SIDDAL dimer.

#### 3.3.3. Metal as Electron Donor

**Figure 14:**
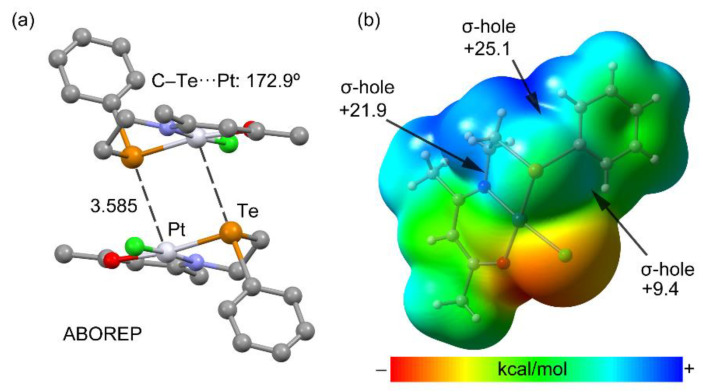
(**a**) Partial view of refcode ABOREP structure. Distances in Å. H-atoms omitted for clarity. (**b**) MEP surface (isodensity 0.001 a.u.) of ABOREP. The energies at the σ-holes are indicated in kcal/mol.

For tellurium, we have also found the interesting Ch···*d*^8^[M] ChB interaction commented above in two structures (M = Pt). Figure 14a shows a self-assembled dimer observed in the solid state of one of both (refcode ABOREP [107]). The Pt is located opposite to the C–Te bond, thus acting as electron donor in the ChB interaction. The MEP surface of chloro-(4-(2-(phenyltelluro)ethylimino)pentan-2-onato-N,O,Te)-platinum(II) complex (see Figure 14b) shows three σ-holes opposite to the three covalent bonds. The σ-hole opposite to the C_aliphatic_–Te bond presents the lowest value (+9.4 kcal/mol) while the other two exhibit similar and significantly larger MEP values (+21.9 and +25.1 kcal/mol) because they are under the influence of the CH bonds.

**Figure 15:**
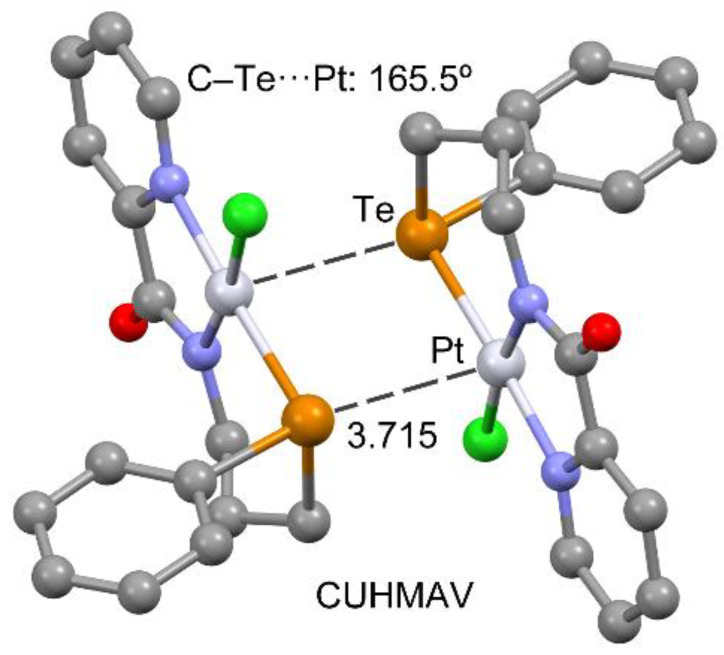
Partial views of the X-ray structures of refcode CUHMAV. Distance in Å. H-atoms omitted for clarity.

The other example of X-ray structure exhibiting Te···Pt ChBs is depicted in Figure 15, that corresponds to chloro-(N-(3-(phenyltellanyl)propyl)pyridine-2-carboxamidato)-platinum(II) complex (refcode CUHMAV [101]). It also forms self-assembled dimers in the solid state where the Pt atom is positioned opposite to the Ar–Te bond (3.715 Å). The directionality (∡C–Te···Pt = 165.5°) of the ChB interactions suggests that the nucleophilic *d*_z_^2^ orbital of Pt is pointing to the tellurium σ-hole, as it has been demonstrated for halogen bonded complexes [97]. Finally, in Table 3, the list of CSD codes including compound names and formulas of the Se involving dimers is given.

## 4. Theoretical Study

### 4.1. Energetic Study

With the purpose to shed light on the nature and directionality of the ChBs studied herein we have performed a computational study on three selected dimers (XILFEE, MELJUJ and CUHMOJ). In Table 4 the binding energies (ΔE_BSSE_) and the contribution of the Chalcogen bond contacts (derived from the V(r) predictor) (ΔE_ChB_), equilibrium distances (d) as well as the value of the density at the bond critical point that characterizes the interaction (ρ·10^2^) are included.

Firstly, the energetics, in all three calculated structures, large and attractive interaction energy values (ranging from −24.5 to −32.0 kcal/mol) were obtained, which involve the chalcogen bonding interactions as well as other noncovalent forces (e.g., hydrogen bonding, metal···metal interactions or π-π stacking). These results agree with very recent reports on similar supramolecular assemblies [108]. Secondly, the chalcogen bonding energies also achieved negative and attractive values (ranging from −2.7 to −7.9 kcal/mol). Finally, the NBO charges analyses resulted in positive values in the case of the ChB donor atom (Se/Te) of around 0.80e and negative ones in the case of the ChB acceptor atom (Cl, O and I), ranging between −0.2 and −0.6e.

### 4.2. AIM Analysis

**Figure 16:**
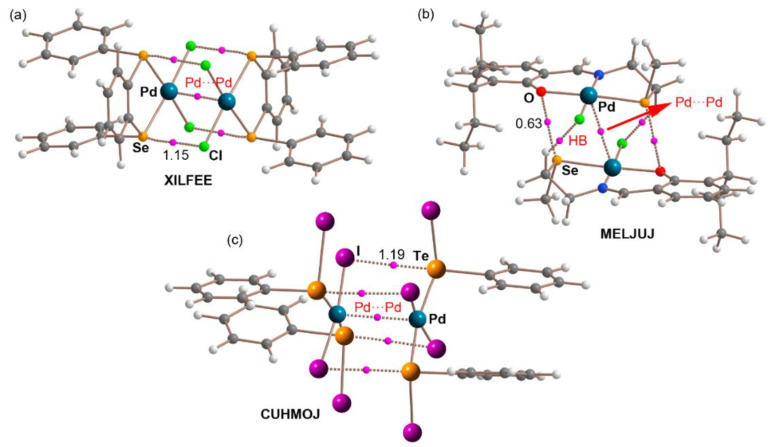
Distribution of intermolecular bond CPs (colored in pink)and bond paths regarding XILFEE (**a**), MELJUJ (**b**) and CUHMOJ (**c**) dimers. The value of the density at the bond CP is given in a.u. (ρ·100). Pd···Pd and ancillary HB interactions are highlighted in red.

Figure 16a shows the QTAIM analysis of the XILFEE self-assembled dimer. The existence of the four Se···Cl ChBs is confirmed by the presence of four bond CPs and bond paths interconnecting the Se and Cl atoms. The QTAIM analysis also confirms the presence of a Pd···Pd interaction, characterized by a bond CP and bond path connecting both atoms. The dimerization energy is very large (−32.0 kcal/mol) due to the coexistence of five contacts, thus validating the structure directing role of such interactions in the solid state.

On the other hand, in Figure 16b the QTAIM analysis of the MELJUJ structure is shown. As noticed, the presence of two Se···O ChBs is confirmed by the existence of two bond CPs and bond paths interconnecting the Se and O atoms. This result differs from the X-ray crystal structure, which exhibited a Se···Pd ChB. In addition, the existence of ancillary hydrogen bonding (HB) interactions between the CH groups from the aliphatic chain of the ligand and the chloride atoms was also confirmed by means of a bond CP and a bond path connecting both moieties. The dimerization energy is also large (−31.5 kcal/mol) due to the existence of both ChB and HB interactions, which contribute to the formation and stabilization of this supramolecular assembly.

Finally, Figure 16c shows the QTAIM analysis of the CUHMOJ self-assembled dimer. The existence of the four Te···I ChBs is corroborated by the presence of four bond CPs and bond paths interconnecting the Te and I atoms. The QTAIM analysis also evidences a Pd···Pd interaction, characterized by a bond CP and bond path connecting both atoms. The dimerization energy is large (−24.5 kcal/mol) due to the coexistence of five contacts, thus further supporting the structure directing role of such interactions in the solid state. The interaction energy is smaller (in absolute value) than that observed for the XILFEE dimer (see Figure 16a) described above, where a similar combination of interactions is observed. This is likely due to the fact that chloride is better electron donor than iodide (the MEP value at the negative belt is larger).

## 5. Concluding Remarks

In this review article, the CSD has been inspected to demonstrate the existence and strong directing role of ChBs interaction involving metal coordinated Se and Te atoms. The formation of the coordination bond has a double effect. First, it provides an additional σ-hole to the Se or Te atoms and, secondly, increases their Lewis base acidity, thus reinforcing their ability to establish ChBs. We have provided several examples of structures retrieved from the CSD that evidence the structure directing role of such interactions. Moreover, the energetic features of the ChBs have been computed and the interactions characterized by using the QTAIM. In some of these examples, the electron donor system is the metal center (Pd or Pt). This result is significant, since Ch···*d*^8^[M] interactions have not been studied before, as far as our knowledge extends. More investigation is thus needed in this direction, since it can be relevant in several fields like supramolecular chemistry, crystal engineering, coordination chemistry and supramolecular catalysis.

## Figures and Tables

**Table 1:** Atomic polarizabilities (α, a.u.) calculated at the MP2/def2-TZVP level of theory of chalcogen (Ch) elements and their van der Waals radii (R_vdW_, Å).

Ch	α	R_vdW_
O	3.0	1.52
S	11.8	1.80
Se	17.5	1.90
Te	25.9	2.06

**Table 2:** List of CSD codes, compound names and formulas of the Se involving dimers.

CSD Code	Name	Formula
DUWMEN	dichloro-(2,3:7,8-bis(methylenedioxy)-selenanthrene-Se,Se′)-platinum(II) acetone solvate	C_14_H_8_Cl_2_O_4_PtSe_2_, C_3_H_6_O
XUFHEM	chloro-(2-(phenyl((2-(phenylselanyl-kSe)ethyl)imino-kN)methyl)phenolato-kO)-palladium(II)	C_21_H_18_ClNOPdSe
XUFHIQ	chloro-(2-(phenyl((2-(phenylselanyl-kSe)ethyl)imino-kN)methyl)phenolato-kO)-platinum(II)	C_21_H_18_ClNOPtSe
XILFEE	(1,2-bis(phenylseleno)benzene)-dichlorido-palladium(II)	C_18_H_14_Cl_2_PdSe_2_
PUYWUC	cichloro-(diphenyl(2-(phenylselanyl)phenyl)phosphine)-platinum	C_24_H_19_Cl_2_PPtSe
TAFWIJ	dichloro-(2-(phenylselanyl)aniline)-palladium(II) acetonitrile solvate	C_12_H_11_Cl_2_NPdSe, C_2_H_3_N
EPULIM	dichloro-(1-(2,6-di-isopropylphenyl)-4-((phenylselanyl)methyl)-1H-1,2,3-triazole)-palladium(II)	C_21_H_25_Cl_2_N_3_PdSe
BEJWIZ	chloro-[8-({[2-(phenylselanyl)ethyl]imino}methyl)naphthalen-1-yl]-palladium(II)	C_19_H_16_ClNPdSe
COKFIT	chloro-(2,4-di-t-butyl-6-(((2-(phenylselanyl)ethyl)imino)methyl)phenolato)-palladium(II)	C_23_H_30_ClNOPdSe
TOQPEV	chloro-(2-(1-(2-(phenylselanyl)ethylimino)ethyl)-1-naphtholato-N,O,Se)-palladium(II)	C_20_H_18_ClNOPdSe
VUGWOK	chloro-(1,7-bis(phenylselenomethyl)-1,7-dicarba-closo-dodecaborate-B,S,S′)-palladium(II)	C_16_H_23_B_10_ClPdSe_2_
QETFED	dichloro-(1-methyl-3-[(phenylselanyl)methyl]-imidazol-2-ylidene)-platinum(II) acetonitrile solvate	C_11_H_12_Cl_2_N_2_PtSe, 0.5(C_2_H_3_N)
QETFAZ	dichloro-(1-methyl-3-[(phenylselanyl)methyl]-imidazol-2-ylidene)-palladium(II) acetonitrile solvate	C_11_H_12_Cl_2_N_2_PdSe, 0.5(C_2_H_3_N)
MELJUJ	chloro-((2-((2-methylselanyl)ethyl)iminomethyl)-6-(1-ethylpropyl)phenolato)-palladium(II)	C_15_H_22_ClNOPdSe

**Table 3:** List of CSD codes, compound names and formulas of the Te involving dimers.

CSD Code	Name	Formula
CUHMOJ	bis(benzenetellurenyl iodide)-di-iodo-palladium(II)	C_12_H_10_I_4_PdTe_2_
CODTEX	dichloro-[1-(2-{[2,6-di-isopropylphenyl]tellanyl}phenyl)-N,N-dimethylmethanamine]-palladium	C_21_H_29_Cl_2_NPdTe
JAGZIA	dibromo-(meso-1,3-bis(phenyltelluro)propane)-palladium(II)	C_15_H_16_Br_2_PdTe_2_
TAPYEO	cis-Dichloro-(2-(4-ethoxyphenyl-telluro)ethyl(methyl)sulfido)-platinum(II)	C_11_H_16_Cl_2_OPtSTe
SIDDAL	chloro-(2-(1-((2-((4-methoxyphenyl)tellanyl)ethyl)amino)ethyl)phenolato)-palladium(II) dichloromethane solvate	C_17_H_20_ClNO_2_PdTe, 0.5(CH_2_Cl_2_)
WIXSEB	(μ_2_-N-(o-phenylene)methyllidene)-N′-((3-formyl-o-phenylene)methylidene)-platinum(II)-tellurium hexafluorophosphate	C_23_H_19_N_2_OPtTe^+^, F_6_P^−^
ABOREP	chloro-(4-(2-(phenyltelluro)ethylimino)pentan-2-onato-N,O,Te)-platinum(II)	C_13_H_16_ClNOPtTe
CUHMAV	chloro-(N-(3-(phenyltellanyl)propyl)pyridine-2-carboxamidato)-platinum(II)	C_15_H_15_ClN_2_OPtTe

**Table 4:** Binding energies and the contribution of the Chalcogen bond contacts (ΔE_BSSE_ and ΔE_ChB_ in kcal/mol, respectively), equilibrium distances (d, in Å), C–Ch···X (X = Cl, O and I), angle (∠, in °) and value of the density at the bond critical point (ρ 10^2^, in a.u.) for XILFEE, MELJUJ and CUHMOJ optimized dimers. The NBO charges of both chalcogen bond donor (Q_D_) and acceptor (Q_A_) atoms are also indicated in e.

CSD Code	ΔE_BSSE_	ΔE_ChB_	d	∠	ρ 10^2^	Q_D_ (e)	Q_A_ (e)
XILFEE	−32.0	−7.9 ^a^	3.359	165.6	1.15	0.78	−0.42
MELJUJ	−31.5	−2.7 ^a^	3.445 ^c^	170.6 ^c^	0.63	0.70	−0.64
CUHMOJ	−24.5	−4.8 ^b^	3.724	178.9	1.19	0.79	−0.15

^a^ Values obtained using the equation E_int_ = 0.375 * V(r) − 0.5655 from reference [86]. ^b^ Values obtained using the equation E_int_ = 0.556 × V(r) + 0.6445 from reference [86]. ^c^ Values measured to the Pd-O bond centroid.

## Data Availability

Crystallographic data used in this study can be obtained from the Cambridge Structural Database (CSD). https://www.ccdc.cam.ac.uk/solutions/csd-core/components/csd/ (accessed on 12 February 2022).

## Supplementary Materials

The following supporting information can be downloaded at: https://www.mdpi.com/article/10.3390/ijms23084188/s1.
