# Supplementary material for: Metal Coordination Enhances Chalcogen Bonds: CSD Survey and Theoretical Calculations
Source: Int J Mol Sci. 2022 Apr 10;23(8):4188. doi: 10.3390/ijms23084188 (PMC9030556; doi:10.3390/ijms23084188)
Supplement: Supplementary file 1 [file ijms-23-04188-s001.zip › ijms-1627430-supplementary.pdf]

International Journal of
Molecular Sciences

MDPI

Metal Coordination Enhances Chalcogen Bonds: CSD Survey and Theoretical Calculations

Antonio Frontera * and Antonio Bauza *

Departament de Química, Universitat de les Illes Balears, Crta. de Valldemossa km 7.5,
07122 Palma de Mllorca, Baleares, Spain
* Correspondence: toni.frontera@uib.es (A.F.); antonio.bauza@uib.es (A.B.)

Results from the CSD search

Table S1. List of X-ray structures exhibiting metal enhanced-Ch bonds including the CSD code, the ChB donor and acceptor atoms, the Ch···X distance d, in Å) and the Y–C···X angle ∠, in°).

CSD code	ChB donor	ChB acceptor	d	∠
HEPXEG	Te	Cl	3.249	174.5
ZEYXIN	Te	Cl	3.309	166.8
OGOXAM	Se	Cl	3.341	174.3
XUFHEM	Se	O	3.353	160.8
ZOWYEP	Te	C	3.581	166.8
BEJWUL	Se	Cl	3.376	168.0
OLURIZ	Te	Cl	3.382	167.6
QATVAK	Te	Cl	3.397	162.8
QADDUU	Te<br>Te	Cl<br>Cl	3.401<br>3.494	174.7
ROXFOB	Te	Cl	3.404	170.9
WICDOD	Se	Cl	3.405	162.4
XILFEE	Se	Cl	3.410	164.7
XUFHIQ	Se	O	3.418	158.9
ZOWYEP10	Te	C	3.583	169.5
QAXKIJ	Te	Cl	3.425	170.3
KEXSAK	Te	Cl	3.429	170.9
XOYTOW	Se	Cl	3.431	167.7
PUYWUC	Se	Cl	3.444	167.8
TOQPEV	Se	C	3.450	157.0
RUHFIM	Te	Cl	3.451	171.1
TAFWIJ	Se	Cl	3.463	176.1
OLUREV	Te	Cl	3.464	166.5
WIXSEB	Te	C	3.465	172.5
DISVIL	Se	Cl	3.470	159.5
UCAJAM	Se	Cl	3.480	160.4
EPULIM	Se	Cl	3.482	161.5
CIPKEU	Se	C	3.484	173.8
KEBXAR	Se	Cl	3.484	164.6
WAVTIY	Se	Cl	3.485	167.0
DEVGUH	S<br>Te	Cl<br>Cl	3.488<br>3.401	167.1
XIJHIJ	Te	Cl<br>Br	3.509<br>3.844	174.2

BEJWIZ	Se	C	3.536	168.6
HIXCEX	Te	Cl	3.539	172.9
WIVZAD	Te	Cl	3.551	167.7
TOTRUR	Te	Cl	3.568	173.8
KEBXEV	Se	Cl	3.575	160.6
ZATMEM	Se	Cl	3.579	176.7
ABOREP	Te	Pt	3.585	173.0
TAPYEO	Te	Cl	3.585	154.7
EKIROG	Se	Cl	3.589	165.0
MULZIC	Se	Cl	3.592	174.6
GOYSIZ	Se	Cl	3.618	152.7
CODTEX	Te	Cl	3.621	154.1
JAGZIA	Te	Br	3.647	152.3
COKFIT	Se	C	3.658	176.2
SIDDAL	Te	C	3.666	166.8
VUGWOK	Se	Pd	3.678	150.7 [a]
PUYXAJ	Se	Cl	3.679	166.7
NEDTAS	Se	Cl	3.681	160.8
QETFED	Se	Pt	3.697	173.6
MELJUJ	Se	Pd	3.701	175.7
SETFON	Se	Cl	3.701	163.8
CODTAT	Te	Cl	3.706	151.3
QETFAZ	Se	Pd	3.708	170.6
CUHMAV	Te	Pt	3.715	166.5
QADDOO	Te	Cl	3.734	175.8
DIZWAL	Te	Cl	3.753	172.3
DISWAE	Se	Cl	3.760	157.7
KICSIA	Se	Br	3.787	169.6
	Se	Cl	3.647	
EKIQUL	Se	Cl	3.795	168.9
PDSECL	Se	Cl	3.820	153.1
LEQSIK	Se	Cl	3.833	166.0
FELPOD	Se	Cl	3.836	159.4
CUHMEZ	Te	Cl	3.850	171.6
YIZPOM	Te	Cl	3.852	153.7
JOZXOL	Te	I	3.862	169.2
BIGDAX	Te	Cl	3.887	160.5
DUWMEN	Se	O	3.909	172.4
CUHMOJ	Te	I	3.937	161.1
REXBOL	Te	Cl	3.937	152.0
WACNIX	Te	Cl	3.989	172.8
UZACEE	Se	I	4.033	165.8

[a] Angle measured to the Cl–Pd bond centroid.

Cartesian coordinates of the calculated X-ray and optimized dimers

DUWMEN

Pt	3.53600000	1.52000000	5.75100000
Se	4.58300000	2.44100000	7.67200000
Se	4.49900000	-0.49500000	6.58600000
Cl	2.57100000	3.53000000	5.12900000
Cl	2.60000000	0.50000000	3.90900000
C	4.01500000	1.11700000	8.84800000
C	3.90600000	-0.17300000	8.33300000
C	3.36600000	-1.26100000	9.24600000
C	3.02500000	-0.94200000	10.41300000
C	3.13100000	0.36000000	10.97900000
C	3.62000000	1.38800000	10.02200000
O	2.50500000	-1.74600000	11.44700000
O	2.66800000	0.45200000	12.29300000
C	2.46800000	-0.94600000	12.70300000
C	6.23800000	1.61100000	7.35200000
C	6.19800000	0.32800000	6.93700000
C	7.46100000	-0.49100000	6.88500000
C	8.57200000	0.24100000	7.10500000
C	8.60900000	1.48800000	7.44000000
C	7.53500000	2.27000000	7.65500000
O	9.89900000	-0.24400000	7.02100000
O	9.85700000	1.92700000	7.60300000
C	10.75700000	0.89600000	7.55700000
Pt	0.96900000	2.75800000	15.17700000
Se	-0.07700000	1.83600000	13.25500000
Se	0.00600000	4.77200000	14.34200000
Cl	1.93400000	0.74700000	15.79800000
Cl	1.90600000	3.77700000	17.01800000
C	0.49000000	3.16000000	12.07900000
C	0.59900000	4.45000000	12.59400000
C	1.13900000	5.53800000	11.68200000
C	1.48000000	5.21900000	10.51400000
C	1.37500000	3.91700000	9.94900000
C	0.88500000	2.88900000	10.90500000
O	2.00000000	6.02300000	9.48000000
O	1.83700000	3.82500000	8.63500000
C	2.03800000	5.22300000	8.22400000
C	-1.73300000	2.66600000	13.57600000
C	-1.69300000	3.94900000	13.99000000
C	-2.95600000	4.76800000	14.04200000

C	-4.06700000	4.03600000	13.82300000
C	-4.10400000	2.79000000	13.48800000
C	-3.03000000	2.00800000	13.27200000
O	-5.39400000	4.52100000	13.90600000
O	-5.35200000	2.35000000	13.32500000
C	-6.25200000	3.38100000	13.37100000
H	3.27840027	-2.27132693	8.90472915
H	3.63950553	2.40925288	10.34068807
H	3.31278433	-1.23315954	13.29357500
H	1.48057790	-1.02563606	13.10742021
H	7.47521911	-1.54362962	6.69350925
H	7.60841207	3.27598663	8.01207361
H	11.04780405	0.65716134	8.55864320
H	11.50158672	1.13062398	6.82526084
H	1.22693011	6.54814378	12.02372772
H	0.86466384	1.86791955	10.58581174
H	3.02583856	5.30212428	7.82049742
H	1.19388764	5.51024923	7.63250849
H	-2.97012360	5.82075583	14.23280273
H	-3.10365230	1.00254634	12.91347765
H	-6.99623576	3.14665536	14.10318559
H	-6.54328159	3.61941303	12.36939416

XUFHEM

Pd	4.06000000	1.52800000	0.73700000
Se	3.07500000	3.59600000	0.17700000
N	4.65800000	2.29100000	2.49800000
O	5.00500000	-0.20100000	1.03300000
Cl	3.21900000	0.68400000	-1.24800000
C	5.33800000	1.64900000	3.39800000
C	5.76600000	2.37900000	4.65300000
C	4.87900000	2.60800000	5.70200000
H	3.99900000	2.31600000	5.64200000
C	5.31300000	3.27100000	6.83500000
H	4.72600000	3.41800000	7.54000000
C	6.59600000	3.71200000	6.92500000
H	6.88000000	4.15200000	7.69300000
C	7.47700000	3.50900000	5.88500000
H	8.35100000	3.82400000	5.94600000
C	7.06400000	2.84000000	4.75600000
H	7.66000000	2.69600000	4.05800000
C	5.74700000	0.24500000	3.29900000

C	6.33400000	-0.35800000	4.43300000
H	6.45600000	0.15700000	5.19800000
C	6.73300000	-1.67000000	4.45300000
H	7.07200000	-2.04400000	5.23500000
C	6.62900000	-2.42900000	3.31000000
H	6.92600000	-3.31000000	3.30700000
C	6.08400000	-1.87800000	2.17400000
H	6.04300000	-2.39400000	1.39900000
C	5.58500000	-0.56500000	2.13900000
C	4.19000000	3.67400000	2.78900000
H	4.80800000	4.09300000	3.40800000
H	3.32300000	3.63000000	3.22100000
C	4.08100000	4.52200000	1.55300000
H	4.97000000	4.72900000	1.22400000
H	3.64100000	5.35800000	1.77300000
C	1.33600000	3.52200000	1.02600000
C	0.80200000	4.64900000	1.60200000
H	1.29700000	5.43500000	1.63500000
C	-0.46800000	4.61200000	2.12500000
H	-0.82100000	5.36900000	2.53400000
C	-1.20900000	3.47700000	2.04700000
H	-2.06500000	3.45800000	2.40900000
C	-0.70000000	2.36500000	1.44400000
H	-1.22300000	1.59800000	1.38200000
C	0.58600000	2.36600000	0.92200000
H	0.93600000	1.60700000	0.51200000
Pd	6.90400000	3.07700000	-0.73700000
Se	7.88900000	1.00900000	-0.17700000
N	6.30600000	2.31400000	-2.49800000
O	5.95900000	4.80600000	-1.03300000
Cl	7.74500000	3.92100000	1.24800000
C	5.62600000	2.95600000	-3.39800000
C	5.19800000	2.22600000	-4.65300000
C	6.08500000	1.99700000	-5.70200000
H	6.96500000	2.28900000	-5.64200000
C	5.65100000	1.33400000	-6.83500000
H	6.23800000	1.18700000	-7.54000000
C	4.36800000	0.89300000	-6.92500000
H	4.08400000	0.45300000	-7.69300000
C	3.48700000	1.09600000	-5.88500000
H	2.61300000	0.78100000	-5.94600000
C	3.90000000	1.76600000	-4.75600000

H	3.30400000	1.90900000	-4.05800000
C	5.21700000	4.36000000	-3.29900000
C	4.63000000	4.96300000	-4.43300000
H	4.50800000	4.44900000	-5.19800000
C	4.23100000	6.27500000	-4.45300000
H	3.89200000	6.64900000	-5.23500000
C	4.33500000	7.03400000	-3.31000000
H	4.03800000	7.91500000	-3.30700000
C	4.88000000	6.48300000	-2.17400000
H	4.92100000	6.99900000	-1.39900000
C	5.37900000	5.17000000	-2.13900000
C	6.77400000	0.93100000	-2.78900000
H	6.15600000	0.51200000	-3.40800000
H	7.64100000	0.97500000	-3.22100000
C	6.88300000	0.08300000	-1.55300000
H	5.99400000	-0.12300000	-1.22400000
H	7.32300000	-0.75200000	-1.77300000
C	9.62800000	1.08300000	-1.02600000
C	10.16200000	-0.04400000	-1.60200000
H	9.66700000	-0.83000000	-1.63500000
C	11.43200000	-0.00700000	-2.12500000
H	11.78500000	-0.76400000	-2.53400000
C	12.17300000	1.12800000	-2.04700000
H	13.02900000	1.14800000	-2.40900000
C	11.66400000	2.24000000	-1.44400000
H	12.18700000	3.00700000	-1.38200000
C	10.37800000	2.23900000	-0.92200000
H	10.02800000	2.99800000	-0.51200000

XUFHIQ

C	5.79700000	6.88300000	4.61900000
Cl	3.23900000	8.62300000	-1.23400000
N	4.67800000	6.97700000	2.47400000
O	4.98300000	9.49700000	1.04100000
Pt	4.05800000	7.74300000	0.74300000
Se	3.07300000	5.68400000	0.19700000
C	7.09500000	6.42100000	4.72400000
H	7.69200000	6.56200000	4.02300000
C	7.51700000	5.75600000	5.85400000
H	8.39100000	5.44600000	5.91600000
C	6.64100000	5.55200000	6.89100000
H	6.93700000	5.10800000	7.65200000

C	5.33700000	5.98800000	6.83100000
H	4.75100000	5.83500000	7.53800000
C	4.91400000	6.66700000	5.68900000
H	4.04000000	6.97900000	5.63500000
C	5.76400000	9.03000000	3.29100000
C	5.58200000	9.84300000	2.14800000
C	6.07200000	11.17100000	2.18200000
H	6.01300000	11.69400000	1.41700000
C	6.62100000	11.70100000	3.30100000
H	6.90600000	12.58600000	3.29500000
C	6.76600000	10.94300000	4.44600000
H	7.12200000	11.31600000	5.22100000
C	6.36800000	9.62800000	4.42100000
H	6.50400000	9.10900000	5.18000000
C	5.36400000	7.62000000	3.38200000
C	4.22200000	5.58300000	2.78200000
H	4.85700000	5.17000000	3.38800000
H	3.36500000	5.62500000	3.23700000
C	4.08600000	4.73700000	1.56900000
H	3.63400000	3.91100000	1.79800000
H	4.96800000	4.51400000	1.22900000
C	1.33200000	5.72000000	1.03900000
C	0.80200000	4.57900000	1.59000000
H	1.30600000	3.79900000	1.62300000
C	-0.49100000	4.59200000	2.09700000
H	-0.85000000	3.81900000	2.46800000
C	-1.23200000	5.73300000	2.05000000
H	-2.08900000	5.74500000	2.41000000
C	-0.72300000	6.84900000	1.48300000
H	-1.24800000	7.61700000	1.44700000
C	0.55700000	6.88300000	0.95500000
H	0.89100000	7.65400000	0.55500000
C	5.17600000	7.01800000	-4.61900000
Cl	7.73400000	5.27800000	1.23400000
N	6.29500000	6.92300000	-2.47400000
O	5.99000000	4.40400000	-1.04100000
Pt	6.91500000	6.15800000	-0.74300000
Se	7.90000000	8.21700000	-0.19700000
C	3.87800000	7.47900000	-4.72400000
H	3.28100000	7.33900000	-4.02300000
C	3.45600000	8.14500000	-5.85400000
H	2.58200000	8.45400000	-5.91600000

C	4.33200000	8.34900000	-6.89100000
H	4.03600000	8.79300000	-7.65200000
C	5.63600000	7.91200000	-6.83100000
H	6.22200000	8.06600000	-7.53800000
C	6.05900000	7.23400000	-5.68900000
H	6.93300000	6.92200000	-5.63500000
C	5.20900000	4.87100000	-3.29100000
C	5.39100000	4.05700000	-2.14800000
C	4.90100000	2.72900000	-2.18200000
H	4.96000000	2.20600000	-1.41700000
C	4.35200000	2.20000000	-3.30100000
H	4.06700000	1.31500000	-3.29500000
C	4.20700000	2.95700000	-4.44600000
H	3.85100000	2.58500000	-5.22100000
C	4.60500000	4.27200000	-4.42100000
H	4.46900000	4.79200000	-5.18000000
C	5.60900000	6.28000000	-3.38200000
C	6.75100000	8.31700000	-2.78200000
H	6.11600000	8.73000000	-3.38800000
H	7.60800000	8.27500000	-3.23700000
C	6.88700000	9.16300000	-1.56900000
H	7.33900000	9.99000000	-1.79800000
H	6.00500000	9.38700000	-1.22900000
C	9.64100000	8.18100000	-1.03900000
C	10.17100000	9.32200000	-1.59000000
H	9.66700000	10.10100000	-1.62300000
C	11.46400000	9.30900000	-2.09700000
H	11.82300000	10.08200000	-2.46800000
C	12.20500000	8.16800000	-2.05000000
H	13.06200000	8.15600000	-2.41000000
C	11.69600000	7.05100000	-1.48300000
H	12.22100000	6.28400000	-1.44700000
C	10.41600000	7.01800000	-0.95500000
H	10.08200000	6.24700000	-0.55500000

XILFEE (X-ray)

Pd	6.66900000	3.68200000	0.03700000
Cl	7.66100000	3.81000000	-2.08600000
Cl	4.76300000	2.59100000	-0.73700000
Se	8.64900000	4.66800000	0.91800000
Se	5.78700000	3.46000000	2.23500000
C	9.67400000	3.05000000	1.30600000

C	10.61700000	3.06300000	2.32900000
H	10.81800000	3.81600000	2.82200000
C	11.30600000	1.88900000	2.61900000
H	11.94700000	1.88600000	3.29400000
C	11.07500000	0.73500000	1.88100000
H	11.51500000	-0.05900000	2.07500000
C	10.17100000	0.75500000	0.83400000
H	10.04200000	-0.04800000	0.32400000
C	9.46200000	1.91600000	0.53400000
H	8.88600000	1.91900000	-0.15700000
C	8.13900000	5.07800000	2.74100000
C	8.97100000	5.89100000	3.50200000
H	9.79000000	6.23700000	3.13600000
C	8.60100000	6.21600000	4.80000000
H	9.14800000	6.72800000	5.29000000
C	7.40100000	5.74800000	5.32800000
H	7.14100000	6.01300000	6.13500000
C	6.56800000	4.94000000	4.56700000
H	5.72400000	4.61300000	4.93600000
C	6.95200000	4.60200000	3.26800000
C	6.57500000	1.74500000	2.73000000
C	7.64700000	1.64500000	3.61500000
H	8.03000000	2.42900000	4.01800000
C	8.16700000	0.38900000	3.90800000
H	8.88800000	0.33300000	4.50300000
C	7.62100000	-0.74900000	3.33000000
H	7.96400000	-1.61800000	3.52000000
C	6.54300000	-0.64200000	2.45900000
H	6.18600000	-1.35800000	2.03500000
C	6.01500000	0.60700000	2.15100000
H	5.33300000	0.66500000	1.54400000
Pd	4.87400000	6.41300000	-0.03700000
Cl	3.88200000	6.28500000	2.08600000
Cl	6.78000000	7.50300000	0.73700000
Se	2.89400000	5.42600000	-0.91800000
Se	5.75600000	6.63400000	-2.23500000
C	1.86900000	7.04500000	-1.30600000
C	0.92700000	7.03200000	-2.32900000
H	0.72500000	6.27900000	-2.82200000
C	0.23700000	8.20600000	-2.61900000
H	-0.40400000	8.20900000	-3.29400000
C	0.46800000	9.36000000	-1.88100000

H	0.02800000	10.15300000	-2.07500000
C	1.37200000	9.33900000	-0.83400000
H	1.50200000	10.14300000	-0.32400000
C	2.08200000	8.17900000	-0.53400000
H	2.65700000	8.17600000	0.15700000
C	3.40400000	5.01700000	-2.74100000
C	2.57200000	4.20300000	-3.50200000
H	1.75300000	3.85800000	-3.13600000
C	2.94300000	3.87900000	-4.80000000
H	2.39500000	3.36600000	-5.29000000
C	4.14200000	4.34600000	-5.32800000
H	4.40200000	4.08100000	-6.13500000
C	4.97500000	5.15400000	-4.56700000
H	5.81900000	5.48200000	-4.93600000
C	4.59100000	5.49200000	-3.26800000
C	4.96800000	8.35000000	-2.73000000
C	3.89600000	8.45000000	-3.61500000
H	3.51300000	7.66600000	-4.01800000
C	3.37600000	9.70500000	-3.90800000
H	2.65500000	9.76200000	-4.50300000
C	3.92200000	10.84400000	-3.33000000
H	3.57900000	11.71300000	-3.52000000
C	5.00000000	10.73600000	-2.45900000
H	5.35700000	11.45300000	-2.03500000
C	5.52800000	9.48800000	-2.15100000
H	6.21000000	9.43000000	-1.54400000

XILFEE (Optimized)

Pd	0.0259097	-1.2736023	-0.9983880
Cl	1.8855076	-0.6085670	-2.2103353
Cl	-1.4901778	-0.4490131	-2.5471537
Se	1.5312272	-2.2668467	0.5708976
Se	-1.8340067	-2.1519639	0.2185603
C	1.6688689	-4.0645805	-0.1555436
C	1.8611430	-5.1412556	0.6987056
H	1.8871089	-4.9981816	1.7723364
C	1.9989538	-6.4155713	0.1701074
H	2.1407845	-7.2580381	0.8373942
C	1.9526470	-6.6105299	-1.2024296
H	2.0533699	-7.6085573	-1.6127543
C	1.7761548	-5.5257401	-2.0476408
H	1.7357849	-5.6701817	-3.1208910

C	1.6365679	-4.2460436	-1.5309849
H	1.4983660	-3.3989765	-2.1927008
C	0.3531964	-2.6905917	2.0351885
C	0.9084784	-2.9754797	3.2739262
H	1.9829997	-2.9303522	3.4061247
C	0.0814838	-3.2781813	4.3442330
H	0.5164040	-3.4906159	5.3134987
C	-1.2957570	-3.2782509	4.1829061
H	-1.9432774	-3.4948267	5.0239681
C	-1.8517935	-2.9643659	2.9529836
H	-2.9275678	-2.9196982	2.8317857
C	-1.0235334	-2.6742820	1.8789038
C	-1.9257474	-3.8893551	-0.6375336
C	-1.6251660	-5.0670086	0.0282113
H	-1.3029370	-5.0534037	1.0610706
C	-1.7194537	-6.2726675	-0.6523646
H	-1.4695605	-7.1931180	-0.1379188
C	-2.1130255	-6.2992532	-1.9808503
H	-2.1782332	-7.2433792	-2.5090510
C	-2.4148946	-5.1127869	-2.6353924
H	-2.7158923	-5.1243392	-3.6765070
C	-2.3248462	-3.9015280	-1.9688808
H	-2.5297789	-2.9707842	-2.4864622
Pd	-0.0259097	1.2736023	0.9983880
Cl	-1.8855076	0.6085670	2.2103353
Cl	1.4901778	0.4490131	2.5471537
Se	-1.5312272	2.2668467	-0.5708976
Se	1.8340067	2.1519639	-0.2185603
C	-1.6688689	4.0645805	0.1555436
C	-1.8611430	5.1412556	-0.6987056
H	-1.8871089	4.9981816	-1.7723364
C	-1.9989538	6.4155713	-0.1701074
H	-2.1407845	7.2580381	-0.8373942
C	-1.9526470	6.6105299	1.2024296
H	-2.0533699	7.6085573	1.6127543
C	-1.7761548	5.5257401	2.0476408
H	-1.7357849	5.6701817	3.1208910
C	-1.6365679	4.2460436	1.5309849
H	-1.4983660	3.3989765	2.1927008
C	-0.3531964	2.6905917	-2.0351885
C	-0.9084784	2.9754797	-3.2739262
H	-1.9829997	2.9303522	-3.4061247

C	-0.0814838	3.2781813	-4.3442330
H	-0.5164040	3.4906159	-5.3134987
C	1.2957570	3.2782509	-4.1829061
H	1.9432774	3.4948267	-5.0239681
C	1.8517935	2.9643659	-2.9529836
H	2.9275678	2.9196982	-2.8317857
C	1.0235334	2.6742820	-1.8789038
C	1.9257474	3.8893551	0.6375336
C	1.6251660	5.0670086	-0.0282113
H	1.3029370	5.0534037	-1.0610706
C	1.7194537	6.2726675	0.6523646
H	1.4695605	7.1931180	0.1379188
C	2.1130255	6.2992532	1.9808503
H	2.1782332	7.2433792	2.5090510
C	2.4148946	5.1127869	2.6353924
H	2.7158923	5.1243392	3.6765070
C	2.3248462	3.9015280	1.9688808
H	2.5297789	2.9707842	2.4864622

BEJWIZ

Pd	4.57600000	2.38800000	31.68700000
Se	2.63800000	2.45400000	30.07400000
N	4.33500000	0.42200000	31.71100000
Cl	4.61200000	4.71600000	31.44400000
C	6.10500000	2.28000000	33.01200000
C	6.44500000	1.10800000	33.73800000
C	2.96900000	1.55600000	27.33200000
H	2.16500000	1.10500000	27.45500000
C	2.25000000	0.58000000	30.39400000
H	1.67900000	0.49400000	31.17300000
H	1.77900000	0.21000000	29.63300000
C	4.74200000	2.95600000	28.18000000
H	5.12400000	3.44700000	28.86800000
C	7.53800000	1.13300000	34.68400000
C	6.89400000	3.38400000	33.22000000
H	6.70700000	4.15500000	32.73500000
C	4.81700000	2.14500000	25.91500000
H	5.25600000	2.07200000	25.09600000
C	4.78400000	-0.40900000	32.57200000
H	4.43600000	-1.27000000	32.53500000
C	3.62400000	1.51800000	26.10700000
H	3.23600000	1.05300000	25.40100000

C	3.50700000	-0.17200000	30.61500000
H	4.02300000	-0.17700000	29.79200000
H	3.29300000	-1.09100000	30.83600000
C	7.18100000	-1.17800000	35.26000000
H	7.40500000	-1.92800000	35.76000000
C	5.36300000	2.88400000	26.93000000
H	6.16000000	3.34300000	26.78500000
C	7.85000000	-0.02500000	35.44100000
H	8.53000000	0.01400000	36.07200000
C	5.78200000	-0.15800000	33.61200000
C	6.16100000	-1.25700000	34.33600000
H	5.72100000	-2.06600000	34.20200000
C	3.53000000	2.27300000	28.36800000
C	8.26000000	2.31900000	34.85400000
H	8.94700000	2.35500000	35.48100000
C	7.96700000	3.41400000	34.11900000
H	8.47700000	4.18700000	34.21300000
Pd	10.15100000	2.38800000	31.68700000
Se	8.21300000	2.45400000	30.07400000
N	9.91000000	0.42200000	31.71100000
Cl	10.18800000	4.71600000	31.44400000
C	11.68000000	2.28000000	33.01200000
C	12.02000000	1.10800000	33.73800000
C	8.54500000	1.55600000	27.33200000
H	7.74000000	1.10500000	27.45500000
C	7.82500000	0.58000000	30.39400000
H	7.25400000	0.49400000	31.17300000
H	7.35400000	0.21000000	29.63300000
C	10.31800000	2.95600000	28.18000000
H	10.69900000	3.44700000	28.86800000
C	13.11400000	1.13300000	34.68400000
C	12.47000000	3.38400000	33.22000000
H	12.28200000	4.15500000	32.73500000
C	10.39200000	2.14500000	25.91500000
H	10.83200000	2.07200000	25.09600000
C	10.35900000	-0.40900000	32.57200000
H	10.01100000	-1.27000000	32.53500000
C	9.19900000	1.51800000	26.10700000
H	8.81100000	1.05300000	25.40100000
C	9.08200000	-0.17200000	30.61500000
H	9.59800000	-0.17700000	29.79200000
H	8.86900000	-1.09100000	30.83600000

C	12.75600000	-1.17800000	35.26000000
H	12.98000000	-1.92800000	35.76000000
C	10.93900000	2.88400000	26.93000000
H	11.73500000	3.34300000	26.78500000
C	13.42500000	-0.02500000	35.44100000
H	14.10500000	0.01400000	36.07200000
C	11.35700000	-0.15800000	33.61200000
C	11.73600000	-1.25700000	34.33600000
H	11.29600000	-2.06600000	34.20200000
C	9.10500000	2.27300000	28.36800000
C	13.83500000	2.31900000	34.85400000
H	14.52200000	2.35500000	35.48100000
C	13.54200000	3.41400000	34.11900000
H	14.05300000	4.18700000	34.21300000

VUGWOK

Pd	2.50000000	4.07600000	15.32800000
Se	0.18500000	4.04400000	14.60500000
Se	4.88100000	4.56200000	15.45200000
Cl	1.99200000	3.80700000	17.69700000
C	0.29200000	4.31500000	12.65100000
H	-0.25300000	3.62700000	12.19500000
H	-0.08100000	5.20300000	12.42300000
C	1.72100000	4.23100000	12.15700000
C	4.25900000	4.74600000	12.67800000
C	5.20300000	5.35600000	13.67500000
H	5.06000000	6.33500000	13.71400000
H	6.13800000	5.19000000	13.39700000
C	-0.40700000	2.21300000	14.79100000
C	-0.52700000	1.29600000	13.75700000
H	-0.34600000	1.55200000	12.86100000
C	-0.91700000	-0.00100000	14.05200000
H	-0.97700000	-0.64300000	13.35400000
C	-1.21900000	-0.37200000	15.34800000
H	-1.48500000	-1.26500000	15.53700000
C	-1.13500000	0.55900000	16.36800000
H	-1.35800000	0.31000000	17.25700000
C	-0.72700000	1.85100000	16.09800000
H	-0.66600000	2.49000000	16.80000000
C	5.07900000	6.14800000	16.54100000
C	4.73400000	6.01300000	17.87800000
H	4.38300000	5.19100000	18.20200000

C	4.90200000	7.08000000	18.73000000
H	4.67700000	6.98600000	19.64800000
C	5.39200000	8.28100000	18.27100000
H	5.47900000	9.01900000	18.86300000
C	5.75400000	8.40300000	16.94700000
H	6.11000000	9.22500000	16.63000000
C	5.60200000	7.33600000	16.07200000
H	5.85600000	7.42200000	15.16000000
B	2.88800000	3.98800000	13.38700000
B	2.48600000	2.70100000	12.23000000
H	1.99000000	1.75700000	12.57000000
B	2.08100000	3.46400000	10.67000000
H	1.31200000	3.02500000	9.98400000
B	2.25000000	5.22100000	10.84700000
H	1.59900000	5.94000000	10.28700000
B	2.75800000	5.53400000	12.50300000
H	2.45500000	6.47100000	13.03500000
B	4.16600000	3.05100000	12.57900000
H	4.81200000	2.34600000	13.16200000
B	3.66600000	2.71300000	10.90700000
H	3.96500000	1.77300000	10.37500000
B	3.52900000	4.27300000	10.04100000
H	3.73400000	4.36500000	8.94500000
B	3.95700000	5.56300000	11.20000000
H	4.45900000	6.51100000	10.87900000
B	4.82600000	4.01500000	11.23700000
H	5.90200000	3.94500000	10.93700000
Pd	-0.79700000	7.48300000	15.46300000
Se	1.51700000	7.51500000	16.18600000
Se	-3.17900000	6.99700000	15.33900000
Cl	-0.29000000	7.75200000	13.09400000
C	1.41000000	7.24300000	18.14000000
H	1.95600000	7.93200000	18.59600000
H	1.78300000	6.35600000	18.36800000
C	-0.01900000	7.32800000	18.63500000
C	-2.55700000	6.81300000	18.11400000
C	-3.50100000	6.20200000	17.11700000
H	-3.35800000	5.22300000	17.07700000
H	-4.43600000	6.36900000	17.39400000
C	2.10900000	9.34500000	16.00000000
C	2.23000000	10.26300000	17.03400000
H	2.04800000	10.00600000	17.93000000

C	2.61900000	11.56000000	16.73900000
H	2.67900000	12.20100000	17.43700000
C	2.92200000	11.93100000	15.44400000
H	3.18700000	12.82300000	15.25400000
C	2.83700000	10.99900000	14.42300000
H	3.06100000	11.24900000	13.53400000
C	2.42900000	9.70700000	14.69300000
H	2.36800000	9.06900000	13.99100000
C	-3.37700000	5.41000000	14.25000000
C	-3.03200000	5.54600000	12.91400000
H	-2.68100000	6.36800000	12.58900000
C	-3.20000000	4.47900000	12.06100000
H	-2.97500000	4.57300000	11.14300000
C	-3.68900000	3.27800000	12.52000000
H	-3.77700000	2.53900000	11.92800000
C	-4.05200000	3.15600000	13.84400000
H	-4.40800000	2.33400000	14.16100000
C	-3.90000000	4.22200000	14.72000000
H	-4.15300000	4.13700000	15.63100000
B	-1.18600000	7.57100000	17.40400000
B	-0.78300000	8.85700000	18.56200000
H	-0.28800000	9.80200000	18.22100000
B	-0.37900000	8.09500000	20.12200000
H	0.39000000	8.53400000	20.80700000
B	-0.54800000	6.33800000	19.94400000
H	0.10400000	5.61900000	20.50400000
B	-1.05500000	6.02400000	18.28800000
H	-0.75200000	5.08800000	17.75600000
B	-2.46400000	8.50700000	18.21200000
H	-3.11000000	9.21200000	17.62900000
B	-1.96400000	8.84600000	19.88400000
H	-2.26300000	9.78600000	20.41600000
B	-1.82700000	7.28500000	20.75000000
H	-2.03200000	7.19400000	21.84600000
B	-2.25500000	5.99500000	19.59200000
H	-2.75700000	5.04800000	19.91300000
B	-3.12400000	7.54300000	19.55400000
H	-4.20000000	7.61400000	19.85400000

MELJUJ (X-ray)

Pd	0.17200000	0.00500000	1.88900000
Cl	0.13600000	-2.29300000	2.22800000

Se	-2.10800000	-0.07000000	1.26400000
O	2.14200000	0.04700000	2.32300000
N	0.10600000	1.97100000	1.62700000
C	2.95200000	1.03600000	2.10500000
C	4.32600000	0.85200000	2.36600000
C	5.20000000	1.86500000	2.08300000
C	4.80500000	3.08700000	1.56400000
C	3.48500000	3.30700000	1.37300000
C	2.51700000	2.32600000	1.63300000
C	1.15300000	2.71400000	1.49800000
C	-1.22200000	2.61400000	1.65200000
C	-2.21000000	1.83000000	0.82500000
C	-3.01400000	-0.03400000	2.98200000
C	4.84200000	-0.43400000	3.02900000
C	4.78500000	-1.63300000	2.09100000
C	5.57200000	-1.49100000	0.80900000
C	4.20800000	-0.70100000	4.36400000
C	4.20800000	0.44500000	5.34100000
H	6.02100000	1.71900000	2.09400000
H	5.46300000	3.63300000	1.38100000
H	3.23700000	4.04800000	1.18400000
H	0.97600000	3.60700000	1.32000000
H	-1.57700000	2.63400000	2.48900000
H	-1.26200000	3.56500000	1.32000000
H	-3.15400000	2.06600000	0.92600000
H	-1.97800000	1.81200000	-0.09100000
H	-3.91500000	0.06800000	2.79200000
H	-2.67100000	0.70300000	3.47500000
H	-2.85000000	-0.74500000	3.33800000
H	3.76090364	-1.81092695	1.83709324
H	6.52600000	-1.38900000	1.09300000
H	5.25000000	-0.79600000	0.28800000
H	5.53800000	-2.23600000	0.18200000
H	4.71200000	-1.33800000	4.79500000
H	3.23500000	-0.87200000	4.20300000
Pd	-0.17200000	-0.00500000	-1.88900000
Cl	-0.13600000	2.29300000	-2.22800000
Se	2.10800000	0.07000000	-1.26400000
O	-2.14200000	-0.04700000	-2.32300000
N	-0.10600000	-1.97100000	-1.62700000
C	-2.95200000	-1.03600000	-2.10500000
C	-4.32600000	-0.85200000	-2.36600000

C	-5.20000000	-1.86500000	-2.08300000
C	-4.80500000	-3.08700000	-1.56400000
C	-3.48500000	-3.30700000	-1.37300000
C	-2.51700000	-2.32600000	-1.63300000
C	-1.15300000	-2.71400000	-1.49800000
C	1.22200000	-2.61400000	-1.65200000
C	2.21000000	-1.83000000	-0.82500000
C	3.01400000	0.03400000	-2.98200000
C	-4.84200000	0.43400000	-3.02900000
C	-4.78500000	1.63300000	-2.09100000
C	-5.57200000	1.49100000	-0.80900000
C	-4.20800000	0.70100000	-4.36400000
C	-4.20800000	-0.44500000	-5.34100000
H	-6.02100000	-1.71900000	-2.09400000
H	-5.46300000	-3.63300000	-1.38100000
H	-3.23700000	-4.04800000	-1.18400000
H	-0.97600000	-3.60700000	-1.32000000
H	1.57700000	-2.63400000	-2.48900000
H	1.26200000	-3.56500000	-1.32000000
H	3.15400000	-2.06600000	-0.92600000
H	1.97800000	-1.81200000	0.09100000
H	3.91500000	-0.06800000	-2.79200000
H	2.67100000	-0.70300000	-3.47500000
H	2.85000000	0.74500000	-3.33800000
H	-3.76090364	1.81092695	-1.83709324
H	-6.52600000	1.38900000	-1.09300000
H	-5.25000000	0.79600000	-0.28800000
H	-5.53800000	2.23600000	-0.18200000
H	-4.71200000	1.33800000	-4.79500000
H	-3.23500000	0.87200000	-4.20300000
H	-5.88004106	0.26565445	-3.22656150
H	-5.15465958	2.48788130	-2.61771602
H	5.15465958	-2.48788130	2.61771602
H	5.88004106	-0.26565445	3.22656150
H	5.08165134	1.04365839	5.18854848
H	3.33434866	1.04365839	5.18854848
H	4.20800000	0.06194061	6.34008233
H	-3.33434866	-1.04365839	-5.18854848
H	-5.08165134	-1.04365839	-5.18854848
H	-4.20800000	-0.06194061	-6.34008233

MELJUJ (Optimized)

Pd	1.0580573	-0.2025476	1.4731645
Cl	0.9897399	-2.5101303	1.5892438
Se	-1.1896430	-0.1395568	2.2101648
O	2.9328207	-0.2818081	0.7620784
N	1.0318993	1.7848564	1.4079388
C	3.6292654	0.7079224	0.3110748
C	4.9567907	0.4392223	-0.1415279
C	5.6857543	1.4601482	-0.7093596
C	5.1977705	2.7656301	-0.8484695
C	3.9523489	3.0499529	-0.3629603
C	3.1566628	2.0516833	0.2349412
C	1.9241770	2.4855781	0.8006122
C	-0.0550564	2.4817251	2.0865448
C	-1.3755193	1.7795249	1.9123058
C	-0.9740411	-0.1506076	4.1427301
C	5.5718796	-0.9332387	0.0005325
C	4.9219029	-1.9825435	-0.9061728
C	4.9206671	-1.6021649	-2.3764554
C	5.6175542	-1.4307270	1.4496173
C	6.2842176	-0.4598910	2.4080337
H	6.6901538	1.2356589	-1.0568583
H	5.8064887	3.5339157	-1.3078764
H	3.5552031	4.0582140	-0.4238380
H	1.7387313	3.5593901	0.7353940
H	0.2017296	2.5483428	3.1499975
H	-0.1377319	3.5039277	1.7008556
H	-2.1394764	2.1625548	2.5878681
H	-1.7276726	1.8540511	0.8832429
H	-1.9409427	0.0503535	4.6023163
H	-0.2313326	0.5822109	4.4495340
H	-0.6305651	-1.1521449	4.3961579
H	3.9051816	-2.1649702	-0.5522988
H	5.9347417	-1.4011556	-2.7349923
H	4.3330177	-0.6967726	-2.5522270
H	4.5099824	-2.4045034	-2.9958342
H	6.1613999	-2.3818887	1.4531715
H	4.6010130	-1.6487061	1.7834122
Pd	-1.0580573	0.2025476	-1.4731645
Cl	-0.9897399	2.5101303	-1.5892438
Se	1.1896430	0.1395568	-2.2101648
O	-2.9328207	0.2818081	-0.7620784
N	-1.0318993	-1.7848564	-1.4079388

C	-3.6292654	-0.7079224	-0.3110748
C	-4.9567907	-0.4392223	0.1415279
C	-5.6857543	-1.4601482	0.7093596
C	-5.1977705	-2.7656301	0.8484695
C	-3.9523489	-3.0499529	0.3629603
C	-3.1566628	-2.0516833	-0.2349412
C	-1.9241770	-2.4855781	-0.8006122
C	0.0550564	-2.4817251	-2.0865448
C	1.3755193	-1.7795249	-1.9123058
C	0.9740411	0.1506076	-4.1427301
C	-5.5718796	0.9332387	-0.0005325
C	-4.9219029	1.9825435	0.9061728
C	-4.9206671	1.6021649	2.3764554
C	-5.6175542	1.4307270	-1.4496173
C	-6.2842176	0.4598910	-2.4080337
H	-6.6901538	-1.2356589	1.0568583
H	-5.8064887	-3.5339157	1.3078764
H	-3.5552031	-4.0582140	0.4238380
H	-1.7387313	-3.5593901	-0.7353940
H	-0.2017296	-2.5483428	-3.1499975
H	0.1377319	-3.5039277	-1.7008556
H	2.1394764	-2.1625548	-2.5878681
H	1.7276726	-1.8540511	-0.8832429
H	1.9409427	-0.0503535	-4.6023163
H	0.2313326	-0.5822109	-4.4495340
H	0.6305651	1.1521449	-4.3961579
H	-3.9051816	2.1649702	0.5522988
H	-5.9347417	1.4011556	2.7349923
H	-4.3330177	0.6967726	2.5522270
H	-4.5099824	2.4045034	2.9958342
H	-6.1613999	2.3818887	-1.4531715
H	-4.6010130	1.6487061	-1.7834122
H	-6.6130426	0.8232125	0.3335756
H	-5.4679603	2.9229386	0.7731907
H	5.4679603	-2.9229386	-0.7731907
H	6.6130426	-0.8232125	-0.3335756
H	7.2991411	-0.2104563	2.0827709
H	5.7242891	0.4758495	2.4771856
H	6.3503885	-0.8833566	3.4128709
H	-5.7242891	-0.4758495	-2.4771856
H	-7.2991411	0.2104563	-2.0827709
H	-6.3503885	0.8833566	-3.4128709

CUHMOJ (X-ray)

Pd	10.24400000	3.52400000	1.08000000
I	10.69200000	1.87500000	-0.87800000
I	9.63400000	5.16200000	2.99900000
Te	10.59200000	1.32300000	2.40900000
I	10.72700000	1.77000000	5.09000000
Te	9.31300000	5.54600000	-0.22900000
I	9.51000000	5.11600000	-2.92700000
C	8.48000000	0.85700000	2.35100000
C	7.64500000	1.23200000	3.38300000
H	7.98900000	1.66200000	4.13300000
C	6.28600000	0.95800000	3.28800000
H	5.71500000	1.21600000	3.97500000
C	5.79000000	0.31500000	2.19900000
H	4.87900000	0.12100000	2.14900000
C	6.62100000	-0.04400000	1.17800000
H	6.27100000	-0.46200000	0.42300000
C	7.98000000	0.21100000	1.25600000
H	8.55100000	-0.05600000	0.56900000
C	7.24500000	5.00700000	-0.17400000
C	6.83700000	3.71700000	0.07100000
H	7.46100000	3.04700000	0.23100000
C	5.46400000	3.42300000	0.08000000
H	5.16700000	2.55300000	0.22400000
C	4.55400000	4.44900000	-0.13000000
H	3.64300000	4.25700000	-0.13500000
C	4.96000000	5.71800000	-0.32700000
H	4.33200000	6.39600000	-0.42200000
C	6.33700000	6.02300000	-0.38800000
H	6.62500000	6.88800000	-0.56900000
Pd	13.35100000	4.91100000	1.08000000
I	12.90200000	6.56000000	-0.87800000
I	13.96000000	3.27400000	2.99900000
Te	13.00200000	7.11300000	2.40900000
I	12.86800000	6.66600000	5.09000000
Te	14.28200000	2.88900000	-0.22900000
I	14.08500000	3.32000000	-2.92700000
C	15.11500000	7.57800000	2.35100000
C	15.95000000	7.20400000	3.38300000
H	15.60500000	6.77400000	4.13300000
C	17.30900000	7.47700000	3.28800000

H	17.88000000	7.21900000	3.97500000
C	17.80400000	8.12000000	2.19900000
H	18.71500000	8.31400000	2.14900000
C	16.97400000	8.47900000	1.17800000
H	17.32300000	8.89800000	0.42300000
C	15.61500000	8.22500000	1.25600000
H	15.04400000	8.49100000	0.56900000
C	16.34900000	3.42800000	-0.17400000
C	16.75800000	4.71900000	0.07100000
H	16.13400000	5.38900000	0.23100000
C	18.13000000	5.01200000	0.08000000
H	18.42700000	5.88300000	0.22400000
C	19.04100000	3.98700000	-0.13000000
H	19.95200000	4.17900000	-0.13500000
C	18.63500000	2.71800000	-0.32700000
H	19.26300000	2.04000000	-0.42200000
C	17.25700000	2.41300000	-0.38800000
H	16.96900000	1.54700000	-0.56900000

CUHMOJ (Optimized)

Pd	-1.6173366	0.0884888	0.0299096
I	-1.8998411	-1.8969146	-1.6461619
I	-1.7485526	2.0829528	1.7119018
Te	-1.9666669	-1.9878964	1.5353405
I	-1.8400411	-1.2526857	4.1666200
Te	-1.8098022	2.1806415	-1.4824030
I	-1.7400411	1.4212186	-4.1111421
C	-4.0877277	-1.8965198	1.4440556
C	-4.7671239	-0.7287318	1.7643792
H	-4.2246954	0.1540373	2.0831850
C	-6.1488883	-0.6981153	1.6641391
H	-6.6803934	0.2144538	1.9081160
C	-6.8445946	-1.8224330	1.2399390
H	-7.9252904	-1.7927070	1.1605231
C	-6.1588703	-2.9840987	0.9182940
H	-6.6989216	-3.8636722	0.5874192
C	-4.7751409	-3.0269437	1.0230182
H	-4.2424739	-3.9364273	0.7688197
C	-3.9236855	2.3258077	-1.4130282
C	-4.7275124	1.1931773	-1.4007959
H	-4.2868790	0.2034263	-1.4347906
C	-6.1042972	1.3386648	-1.3418031

H	-6.7314163	0.4550135	-1.3259292
C	-6.6724896	2.6042706	-1.2917358
H	-7.7500205	2.7127592	-1.2437066
C	-5.8641838	3.7312702	-1.3040205
H	-6.3052834	4.7206255	-1.2652272
C	-4.4845335	3.5958952	-1.3677071
H	-3.8556350	4.4792063	-1.3772085
Pd	1.6173366	-0.0884888	0.0299096
I	1.8998411	1.8969146	-1.6461619
I	1.7485526	-2.0829528	1.7119018
Te	1.9666669	1.9878964	1.5353405
I	1.8400411	1.2526857	4.1666200
Te	1.8098022	-2.1806415	-1.4824030
I	1.7400411	-1.4212186	-4.1111421
C	4.0877277	1.8965198	1.4440556
C	4.7671239	0.7287318	1.7643792
H	4.2246954	-0.1540373	2.0831850
C	6.1488883	0.6981153	1.6641391
H	6.6803934	-0.2144538	1.9081160
C	6.8445946	1.8224330	1.2399390
H	7.9252904	1.7927070	1.1605231
C	6.1588703	2.9840987	0.9182940
H	6.6989216	3.8636722	0.5874192
C	4.7751409	3.0269437	1.0230182
H	4.2424739	3.9364273	0.7688197
C	3.9236855	-2.3258077	-1.4130282
C	4.7275124	-1.1931773	-1.4007959
H	4.2868790	-0.2034263	-1.4347906
C	6.1042972	-1.3386648	-1.3418031
H	6.7314163	-0.4550135	-1.3259292
C	6.6724896	-2.6042706	-1.2917358
H	7.7500205	-2.7127592	-1.2437066
C	5.8641838	-3.7312702	-1.3040205
H	6.3052834	-4.7206255	-1.2652272
C	4.4845335	-3.5958952	-1.3677071
H	3.8556350	-4.4792063	-1.3772085

SIDDAL

Te	4.40900000	5.34200000	11.41900000
Te	8.47700000	7.18400000	13.42400000
Pd	4.35200000	6.92800000	13.35100000
Pd	6.98100000	5.50900000	14.50300000

Cl	5.67900000	8.39800000	12.15500000
Cl	7.25300000	4.10500000	12.69200000
O	-0.67500000	7.64200000	8.57400000
O	4.43100000	8.13700000	15.01000000
O	13.59600000	5.40200000	16.55900000
O	5.81400000	4.07200000	15.36900000
N	3.01500000	5.70700000	14.33600000
H	3.48200000	5.34600000	15.05500000
N	6.76500000	6.79200000	16.10100000
H	5.93900000	7.19900000	15.97000000
C	2.60600000	6.01500000	10.50600000
C	1.62200000	5.16900000	10.04900000
H	1.70900000	4.23200000	10.18000000
C	0.49500000	5.67100000	9.38900000
H	-0.18400000	5.08000000	9.08200000
C	0.37500000	7.03500000	9.18800000
C	1.39000000	7.87600000	9.61900000
H	1.32100000	8.81100000	9.46300000
C	2.49300000	7.37700000	10.26400000
H	3.18300000	7.96700000	10.54800000
C	-1.63400000	6.82100000	7.89900000
H	-1.18700000	6.30500000	7.19600000
H	-2.32400000	7.38800000	7.49500000
H	-2.04900000	6.20800000	8.54000000
C	3.51800000	3.88400000	12.72500000
H	3.10300000	3.16100000	12.19000000
H	4.21400000	3.48100000	13.30300000
C	2.48000000	4.55100000	13.56900000
H	1.74200000	4.86300000	12.98700000
H	2.10500000	3.89000000	14.20400000
C	1.92200000	6.52700000	14.92900000
H	1.23500000	5.89400000	15.28700000
C	1.24300000	7.38000000	13.86200000
H	1.85300000	8.09200000	13.57500000
H	0.42800000	7.77900000	14.23200000
H	1.01300000	6.82000000	13.09300000
C	2.39100000	7.38000000	16.08200000
C	3.56400000	8.15300000	16.02400000
C	3.85400000	9.00300000	17.10400000
H	4.64500000	9.52900000	17.08500000
C	3.01200000	9.08800000	18.19300000
H	3.21400000	9.68700000	18.90200000

C	1.87400000	8.30300000	18.25100000
H	1.30100000	8.34500000	19.00800000
C	1.58000000	7.46300000	17.21000000
H	0.80000000	6.92100000	17.26000000
C	10.21900000	6.58100000	14.48500000
C	11.02600000	7.49900000	15.12500000
H	10.80700000	8.42200000	15.10100000
C	12.16000000	7.07100000	15.80000000
H	12.71900000	7.70300000	16.23700000
C	12.48000000	5.71900000	15.84000000
C	11.66700000	4.78800000	15.19500000
H	11.88000000	3.86200000	15.22100000
C	10.54600000	5.23100000	14.51300000
H	9.99400000	4.60500000	14.06200000
C	14.04000000	4.05500000	16.51900000
H	13.35600000	3.47400000	16.91700000
H	14.87400000	3.97200000	17.02900000
H	14.19500000	3.78900000	15.59000000
C	7.91800000	8.62100000	14.90500000
H	8.62400000	9.30900000	14.98900000
H	7.07700000	9.06900000	14.63700000
C	7.72900000	7.92000000	16.22000000
H	8.60100000	7.57200000	16.53500000
H	7.39600000	8.56600000	16.89100000
C	6.62500000	6.05400000	17.38600000
H	6.57300000	6.73100000	18.11900000
C	7.85800000	5.17200000	17.65400000
H	7.88400000	4.43900000	17.00300000
H	7.80600000	4.80300000	18.55900000
H	8.67100000	5.71300000	17.56800000
C	5.35500000	5.24400000	17.42800000
C	5.08600000	4.26600000	16.45300000
C	3.96300000	3.43500000	16.65300000
H	3.77300000	2.74900000	16.02200000
C	3.13000000	3.59900000	17.75200000
H	2.38300000	3.02300000	17.87000000
C	3.37900000	4.59000000	18.67300000
H	2.80300000	4.71400000	19.41600000
C	4.47800000	5.40100000	18.49700000
H	4.64400000	6.09000000	19.12800000
Te	6.86500000	7.81800000	8.55800000
Te	2.79800000	5.97600000	6.55400000

Pd	6.92300000	6.23200000	6.62600000
Pd	4.29400000	7.65100000	5.47400000
Cl	5.59500000	4.76200000	7.82200000
Cl	4.02200000	9.05500000	7.28600000
O	11.95000000	5.51800000	11.40300000
O	6.84300000	5.02300000	4.96700000
O	-2.32100000	7.75800000	3.41800000
O	5.46000000	9.08800000	4.60800000
N	8.26000000	7.45300000	5.64200000
H	7.79200000	7.81400000	4.92200000
N	4.51000000	6.36800000	3.87700000
H	5.33500000	5.96100000	4.00700000
C	8.66800000	7.14500000	9.47100000
C	9.65300000	7.99100000	9.92900000
H	9.56500000	8.92800000	9.79700000
C	10.78000000	7.48900000	10.58800000
H	11.45800000	8.08000000	10.89600000
C	10.90000000	6.12500000	10.79000000
C	9.88500000	5.28400000	10.35800000
H	9.95400000	4.34900000	10.51400000
C	8.78100000	5.78300000	9.71300000
H	8.09200000	5.19300000	9.42900000
C	12.90900000	6.33900000	12.07800000
H	12.46100000	6.85500000	12.78100000
H	13.59800000	5.77200000	12.48200000
H	13.32400000	6.95200000	11.43700000
C	7.75700000	9.27600000	7.25200000
H	8.17100000	9.99900000	7.78700000
H	7.06100000	9.67900000	6.67400000
C	8.79500000	8.60900000	6.40900000
H	9.53200000	8.29700000	6.99000000
H	9.16900000	9.27000000	5.77300000
C	9.35200000	6.63300000	5.04800000
H	10.04000000	7.26600000	4.69100000
C	10.03200000	5.78000000	6.11500000
H	9.42100000	5.06800000	6.40300000
H	10.84700000	5.38100000	5.74500000
H	10.26100000	6.34000000	6.88400000
C	8.88400000	5.78000000	3.89600000
C	7.71100000	5.00700000	3.95300000
C	7.42000000	4.15700000	2.87300000
H	6.62900000	3.63100000	2.89300000

C	8.26200000	4.07200000	1.78400000
H	8.06000000	3.47300000	1.07500000
C	9.40000000	4.85700000	1.72600000
H	9.97300000	4.81500000	0.96900000
C	9.69500000	5.69700000	2.76700000
H	10.47400000	6.23900000	2.71700000
C	1.05500000	6.57900000	5.49200000
C	0.24800000	5.66100000	4.85200000
H	0.46700000	4.73800000	4.87600000
C	-0.88500000	6.08900000	4.17700000
H	-1.44400000	5.45700000	3.74000000
C	-1.20500000	7.44100000	4.13700000
C	-0.39200000	8.37200000	4.78300000
H	-0.60500000	9.29800000	4.75700000
C	0.72900000	7.92900000	5.46400000
H	1.28100000	8.55500000	5.91500000
C	-2.76500000	9.10500000	3.45800000
H	-2.08100000	9.68600000	3.06100000
H	-3.60000000	9.18800000	2.94900000
H	-2.92100000	9.37100000	4.38700000
C	3.35700000	4.53900000	5.07200000
H	2.65000000	3.85100000	4.98800000
H	4.19800000	4.09100000	5.34000000
C	3.54500000	5.24000000	3.75800000
H	2.67300000	5.58800000	3.44200000
H	3.87800000	4.59400000	3.08600000
C	4.65000000	7.10600000	2.59100000
H	4.70100000	6.42900000	1.85800000
C	3.41700000	7.98800000	2.32300000
H	3.39100000	8.72100000	2.97500000
H	3.46900000	8.35700000	1.41800000
H	2.60300000	7.44700000	2.40900000
C	5.91900000	7.91600000	2.54900000
C	6.18800000	8.89400000	3.52400000
C	7.31200000	9.72500000	3.32400000
H	7.50200000	10.41100000	3.95500000
C	8.14400000	9.56100000	2.22500000
H	8.89100000	10.13700000	2.10800000
C	7.89500000	8.57000000	1.30500000
H	8.47200000	8.44600000	0.56100000
C	6.79600000	7.75900000	1.48000000
H	6.63100000	7.07000000	0.84900000

ABOREP

Pt	12.30300000	2.63900000	-0.07400000
Te	12.11800000	3.91800000	2.07200000
Cl	10.20800000	3.42700000	-0.74800000
O	12.50800000	1.83500000	-1.94300000
N	13.99500000	1.83800000	0.61700000
C	10.90400000	2.64600000	3.30200000
C	9.78900000	2.08500000	2.82700000
H	9.55500000	2.19900000	1.93500000
C	8.98600000	1.33900000	3.65700000
H	8.19400000	0.95800000	3.33700000
C	9.34200000	1.15900000	4.96800000
H	8.80700000	0.64000000	5.52200000
C	10.41000000	1.69000000	5.45900000
H	10.62200000	1.54500000	6.35400000
C	11.28300000	2.49000000	4.66900000
H	12.04600000	2.89000000	5.02000000
C	14.00000000	3.14800000	2.70700000
H	14.71100000	3.75300000	2.44600000
H	14.01300000	3.05900000	3.67300000
C	14.20900000	1.77400000	2.04900000
H	15.11100000	1.46700000	2.22600000
H	13.59300000	1.13400000	2.43700000
C	14.84800000	1.21900000	-0.21600000
C	16.11400000	0.54100000	0.35700000
H	16.74300000	0.38600000	-0.35300000
H	15.87500000	-0.29700000	0.76000000
H	16.51200000	1.11300000	1.01700000
C	14.70900000	1.09700000	-1.60400000
H	15.44100000	0.75500000	-2.06400000
C	13.62100000	1.42300000	-2.35400000
C	13.67900000	1.22700000	-3.86300000
H	12.92700000	1.66200000	-4.27200000
H	13.65400000	0.28900000	-4.06500000
H	14.49200000	1.60600000	-4.20400000
Pt	13.83500000	6.34900000	0.07400000
Te	14.02000000	5.07000000	-2.07200000
Cl	15.93000000	5.56100000	0.74800000
O	13.63000000	7.15300000	1.94300000
N	12.14300000	7.15000000	-0.61700000
C	15.23400000	6.34200000	-3.30200000

C	16.34900000	6.90300000	-2.82700000
H	16.58300000	6.78900000	-1.93500000
C	17.15200000	7.64900000	-3.65700000
H	17.94400000	8.03000000	-3.33700000
C	16.79600000	7.82900000	-4.96800000
H	17.33100000	8.34800000	-5.52200000
C	15.72800000	7.29800000	-5.45900000
H	15.51600000	7.44300000	-6.35400000
C	14.85500000	6.49800000	-4.66900000
H	14.09200000	6.09800000	-5.02000000
C	12.13800000	5.84000000	-2.70700000
H	11.42700000	5.23500000	-2.44600000
H	12.12500000	5.92900000	-3.67300000
C	11.92900000	7.21400000	-2.04900000
H	11.02700000	7.52100000	-2.22600000
H	12.54500000	7.85400000	-2.43700000
C	11.29000000	7.76900000	0.21600000
C	10.02400000	8.44700000	-0.35700000
H	9.39500000	8.60200000	0.35300000
H	10.26300000	9.28500000	-0.76000000
H	9.62600000	7.87500000	-1.01700000
C	11.42900000	7.89100000	1.60400000
H	10.69700000	8.23300000	2.06400000
C	12.51700000	7.56500000	2.35400000
C	12.45900000	7.76100000	3.86300000
H	13.21100000	7.32600000	4.27200000
H	12.48400000	8.69900000	4.06500000
H	11.64600000	7.38200000	4.20400000
